# Cannabidiol suppresses proliferation and induces cell death, autophagy and senescence in human cholangiocarcinoma cells via the PI3K/AKT/mTOR pathway

**DOI:** 10.1016/j.jtcme.2024.04.007

**Published:** 2024-04-17

**Authors:** Thatsanapong Pongking, Kitti Intuyod, Phonpilas Thongpon, Raynoo Thanan, Chutima Sitthirach, Apisit Chaidee, Suppakrit Kongsintaweesuk, Sirinapha Klungsaeng, Nuttanan Hongsrichan, Chadamas Sakonsinsiri, Kulthida Vaeteewoottacharn, Somdej Kanokmedhakul, Somchai Pinlaor, Porntip Pinlaor

**Affiliations:** a Biomedical Sciences Program, Graduate School, Khon Kaen University, Khon Kaen 40002, Thailand; b Department of Pathology, Faculty of Medicine, Khon Kaen University, Khon Kaen 40002, Thailand; c Department of Parasitology, Faculty of Medicine, Khon Kaen University, Khon Kaen 40002, Thailand; d Department of Biochemistry, Faculty of Medicine, Khon Kaen University, Khon Kaen 40002, Thailand; e Centre for Research and Development of Medical Diagnostic Laboratories, Faculty of Associated Medical Sciences, Khon Kaen University, Khon Kaen 40002, Thailand; f Department of Chemistry, Faculty of Science, Khon Kaen University, Khon Kaen 40002, Thailand; g Cholangiocarcinoma Research Institute, Faculty of Medicine, Khon Kaen University, Khon Kaen 40002, Thailand

**Keywords:** Cholangiocarcinoma, Cannabidiol, Cannabis, Autophagy, Cellular senescence, Cell cycle arrest

## Abstract

**Background and aim:**

Cholangiocarcinoma (CCA) is usually diagnosed at a late stage, leading to treatment failure. Cannabidiol (CBD), exhibits diverse anti-cancer effects in various cancers, offering avenues for improving CCA treatment. This study investigated the effects of CBD on human CCA cells and the underlying mechanisms *in vitro* and *in vivo*.

**Experimental procedure:**

The effects of CBD on three CCA cell lines (KKU-213B, KKU-100, KKU-055) were assessed using the SRB assay, clonogenic assay, cell cycle arrest, and 3D holotomography. Morphological changes were examined using transmission electron microscopy, while mitochondrial ROS levels and mitochondrial membrane potential were studied using MitoSOX, JC-1, and DCFH-DA. Cellular senescence induction was evaluated via SA-β-gal staining. Protein associatedwith autophagy and cellular senescence were analyzed using Western blot and/or immunofluorescent assays. A xenograft model demonstrated the anti-tumor activity of CBD and the induction of cellular senescence through immunohistochemistry targeting PCNA, β-gal, and p21.

**Results and conclusion:**

CBD effectively inhibited CCA cell proliferation, suppressed colony formation and induced G0/G1 phase cell cycle arrest. Morphological examination revealed lipid droplets/vesicles in CCA cell lines. CBD induced autophagy by upregulating LC3BII, downregulating p62, and inhibiting the *p*-PI3K, *p*-AKT, and *p*-mTOR pathways. Additionally, CBD disrupted mitochondrial homeostasis by elevating ROS, reducing membrane potential, and induced cellular senescence by increasing the expression of p53 and p21. *In-vitro* results were confirmed by xenograft models. Overall, CBD suppresses proliferation and induces cell death, autophagy and senescence in CCA cells via the PI3K/AKT/mTOR pathway, which indicates a therapeutic option for CCA treatment.

## Graphical abstract

**Figure:**
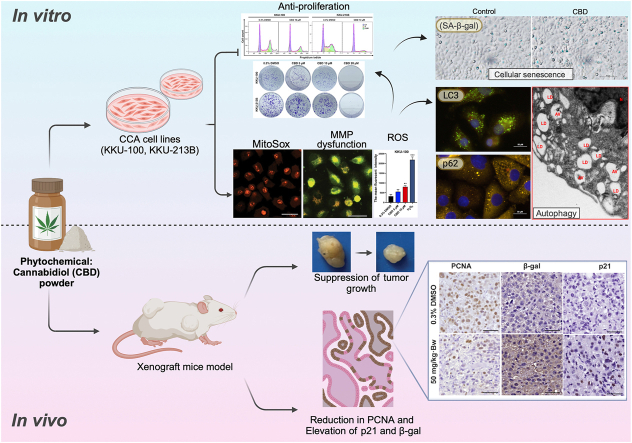

## 1 Introduction

The incidence of cholangiocarcinoma (CCA), commonly known as bile-duct cancer, is increasing worldwide. There is a high prevalence of CCA in the Greater Mekong subregion, primarily due to infections with liver flukes, notably *Opisthorchis viverrini* and *Clonorchis sinensis*. The reported incidence rates of CCA in the region exceed 6 per 100,000 population.^1^ Unfortunately, most CCA patients are diagnosed at an advanced stage, resulting in a poor prognosis, unsatisfactory surgical outcomes, and low survival rates.^1^ Chemotherapy plays a central role in CCA treatment, typically involving drugs such as gemcitabine (GEM), 5-fluorouracil, and cisplatin.^2^ However, the effectiveness of chemotherapy decreases over time due to cancer adaptation.^3^^,^^4^ Although the combination of GEM with other anti-cancer drugs has shown improved response rates in CCA, it is associated with the risk of adverse effects such as hepatotoxicity, nausea, decreased immunity, diarrhea, and sensory neuropathy.^5^ Therefore, there is an urgent need for alternative therapeutic approaches to improve efficacy of CCA treatment.^1^

Cannabis is derived from a plant scientifically known as *Cannabis sativa* L.^6^ Throughout history, cannabis has been used for the treatment of various diseases, including multiple sclerosis,^7^ and end-stage cancer.^8^ Numerous cannabis-derived phytochemicals, such as cannabidiol (CBD), delta-9-tetrahydrocannabinol (Δ^9^-THC), cannabinol (CBN), cannabigerol (CBG), and cannabichromene (CBC), have shown potential therapeutic effects in clinical trials for a variety of diseases, including cancer.^9^ Compelling evidence suggests that CBD can induce cancer-cell death through apoptosis and autophagy in various cancer types.^10^, ^11^, ^12^

Previous studies have highlighted the critical role of the PI3K/AKT/mTOR pathway in cancer progression, particularly in *O. viverrini*-associated CCA.^13^ Activation of this pathway elicits several dynamic changes, including cell cycle arrest, apoptosis, autophagy, reduction in mitochondrial membrane potential, and an excessive generation of reactive oxygen species (ROS). These changes contribute to lipid droplet accumulation or vesicle formation.^14^ In addition, it has been shown that inhibiting the PI3K/AKT/mTOR pathway induces cellular senescence in various cancers.^15^ Hence, the use of cannabis-derived phytochemicals, particularly CBD, presents a promising approach to suppressing CCA cells through the modulation of multiple signaling pathways, thereby potentially minimizing undesired side effects.

This study aimed to gain a better understanding of the effects of CBD on CCA and its molecular mechanisms, using three human CCA cell lines (KKU-055, KKU-100, and KKU-213B). It is hypothesized that CBD exerts its anti-CCA effects through a variety of mechanisms, with some involvement of the PI3K/AKT/mTOR. The efficacy of CBD in combating CCA was also validated through *in vivo* experiments. The results obtained from this study provide compelling evidence supporting the potential inclusion of CBD in clinical trials as a therapeutic option for CCA treatment.

## 2 Materials and methods

### 2.1 CCA cell culture and cannabidiol (CBD) preparation

The CCA cell lines (KKU-055, KKU-100, and KKU-213B) were originally established from patients with opisthorchiasis-associated CCA by Sripa et al.^16^ These CCA cell lines were cultured in Dulbecco's modified Eagle medium (DMEM) supplemented with 4.5 g/L glucose (Gibco, Grand Island, NY, USA), 10 % fetal bovine serum (FBS; Corning, Woodland, CA,USA), 100 U/ml penicillin and 100 μg/ml streptomycin (Gibco) at 37 °C with 5 % CO_2_ in a humidified incubator.

The CBD (#02A64217) used in this study was obtained from the Department of Medical Sciences Reference Standards, Bureau of Drug and Narcotic, Department of Medical Sciences, Ministry of Public Health, Nonthaburi, Bangkok, Thailand. The CBD stock solution was prepared by dissolving it in dimethyl sulfoxide (DMSO; PanReac AppliChem ITW Reagents, Darmstadt, Germany) and diluted with complete medium to make 2–20 μM final concentration.

### 2.2 Cell proliferation detection by sulforhodamine B (SRB) assay

The SRB assay was slightly modified as previously described.^17^ Cells (2 × 10^3^ cells/well) were seeded into flat-bottom 96-well plates and treated with various concentrations of CBD for 24, 48 and 72 h. Subsequently, cells were fixed with 40 % cold trichloroacetic acid for 1 h and stained with 0.4 % (w/v) SRB solution in 1 % acetic acid for 1 h. SRB dye was dissolved in 10 mM Tris buffer pH 10.5. Absorbance was measured at 492 nm using an ELISA reader (Tecan group Ltd., Männedorf, Switzerland).

### 2.3 Clonogenicity assay

CCA cells (1 × 10^3^ cells/well) were grown in 6-well plates and treated with various concentrations of CBD. The culture medium was changed every 2 days, and cells were grown for approximately 2 weeks. Subsequently, cells were stained, and absorbance was measured as previously described.^17^

### 2.4 Cell cycle analysis

CCA cells (2 × 10^5^ cells/well) were seeded into 6-well plates and treated with 10 μM CBD for 48 h. Subsequently, cells were fixed with 70 % ethanol and stained with FxCycle™ PI/RNase staining solution as described.^17^ The stained cells were then sorted and analyzed using a BD FACSCanto II flow cytometer (BD Biosciences, San Jose, CA, USA) and FlowJo™ software version 10.8.1 (Becton, Dickinson & Company, OR, USA).

### 2.5 Lipid droplet/vesicle formation

Lipid droplet/vesicle formation was assessed as previously mentioned.^18^ CCA cells (2 × 10^5^ cells/well) were seeded into 6-well plates overnight. Then, cells were treated with 10 μM CBD for 48 h. A Nikon Eclipse TS 100 inverted light microscope (Nikon, Tokyo, Japan) was used to visualize cell morphology.

### 2.6 Optical diffraction tomography (ODT) and fluorescence imaging

To localize the lipid droplet accumulation and vesicle formation, ODT and fluorescence imaging were performed as previously described.^19^ CCA cells were exposed to 10 μM CBD for 48 h and cultured in a TomoDish at a density of 2 × 10^4^ cells per dish. Live cells were imaged using a Mach-Zehnder interferometric microscope HT-2H (Tomocube, Inc., Daejeon, Republic of Korea). Fluorescence imaging was used for additional 3D visualization, reconstructed via deconvolution using TomoStudio software (Tomocube, Inc.).

### 2.7 Transmission electron microscopy (TEM)

CCA cells (2 × 10^5^ cells/well) were seeded in a 6-well plates and treated with 10 μM CBD for 48 h. Cells were processed according to the methods outlined in a previous article.^20^ TEM photographs were obtained using a JEOL JEM 1011 microscope, equipped with a Morada camera (JEOL Ltd., Tokyo, Japan).

### 2.8 Mitochondrial ROS assay

CCA cells (2.5 × 10^4^ cells/well) were seeded onto 8-well chamber slides. After overnight incubation, cells were treated with 10 μM CBD for 48 h. Then, cells were washed with 1X PBS, exposed to 5 μM MitoSOX Red for 30 min at RT, followed by Hoechst 33,342 (#H3570, Invitrogen).^21^ Imaging was conducted using Zeiss LSM880 confocal scanning microscope, capturing ten fields of view/group (20 × magnification). Fluorescence intensity was analyzed using ImageJ software as previously described.^22^

### 2.9 Mitochondrial membrane potential (MMP; ΔΨm) assay

The MMP assay was performed as previously described.^23^ CCA cells were seeded in 96-well plates (1 × 10^4^ cells/well) and treated with 5 and 10 μM CBD for 48 h. A positive control was exposed to 50 μM carbonyl cyanide *m*-chlorophenyl hydrazone (CCCP) for 10 min. JC-1 dye (200 μM) was added to each well and incubated for 20 min. Confocal images were captured using a Zeiss LSM880 microscope with ten fields per experimental group at 20 × magnification. Fluorescence intensity was quantified using ImageJ software, and mean values were calculated with GraphPad Prism 9.0 (GraphPad Software, La Jolla, CA, USA).

### 2.10 Reactive oxygen species (ROS) detection

CCA cells (2 × 10^5^ cells/well) were seeded into 6-well plates. After overnight incubation, cells were treated with 10 μM CBD for 12–24 h and 1 mM hydrogen peroxide (H_2_O_2_) as a positive control for 3 h. Following treatment, cells were incubated with 25 μM 2′,7′-dichlorofluorescein diacetate (DCFH-DA) for 30 min in the dark,^24^ and washed twice with 1X PBS. Subsequently, cells were transferred to a FACS tube, washed twice with 1X PBS, and analyzed for ROS production using a flow cytometer (BD FACSCanto™ II) with data analyzed using BDFACSDiva™ software.

### 2.11 Senescence-associated β-galactosidase (SA- β-gal) staining

The effect of CBD on cellular senescence in CCA cells was investigated by SA-β-gal staining as previously described.^25^ Cells were seeded into 6-well plates and treated with CBD for 48 h, and then stained with senescence β-galactosidase staining kit (#9860, Cell Signaling Technology, Danvers, MA, USA) following the manufacturer's guidelines. Stained cells were imaged using a light microscope with ten fields per well captured at 20× magnification. Scoring of stained cells was done with the experiment replicated twice independently, each with triplicate technical replicates. Percentage of SA-β-gal-positive cells relative to total cells was computed for quantification.

### 2.12 Immunofluorescence assay (IFA)

CCA cells (2.5 × 10^4^ cells/well) were systematically seeded onto sterile cover slides within 24-well plates and treated with 10 μM CBD for 48 h. Additionally, a subset of cells was pre-treated with chloroquine (CQ) at a concentration of 10 μM, administered 8 h prior to CBD exposure.^26^^,^^27^ After fixing with 4 % paraformaldehyde and permeabilizing with 0.2 % Triton X-100 (Amresco, Solon, OH, USA), cells were blocked with PBS-5% BSA with 0.1 % Triton X-100 and incubated overnight with primary antibodies against LC3BI/II (1:250, #A7198, ABclonal, Wuhan, China) and p62 (1:250, #A7758, ABclonal). Subsequently, cells underwent incubation with secondary antibodies (1:10, #5230-0298, KPL Affinity Purified Antibody Fluorescein-Labeled Goat Anti-Rabbit IgG (H + L), SeraCare Life Sciences Inc., MA, USA). Concurrently, CellMask Deep Red, a specialized plasma membrane stain (#C10046, Invitrogen), was employed to highlight the cellular plasma membranes. Additionally, Hoechst 33,342 was applied for nuclear counterstaining purposes. Ten fields from images of each experimental group were acquired at a magnification of 100 × using a fluorescence imaging microscope (ECLIPSE Ni–U, Nikon Instruments Inc, Japan). Subsequently, fluorescence-intensity images were analyzed using ImageJ software^22^ (National Institutes of Health, Bethesda, MD, USA).

### 2.13 Western blotting

Protein extraction was performed using 1X RIPA reagent (#9806S, Cell Signaling Technology) and protein concentrations were determined using Pierce BCA Protein Assay Kit (Thermo Fisher Scientific Inc., Rockford, IL, USA). SDS-PAGE technique was used to separate proteins (20–25 μg).^27^ PVDF membranes were incubated with primary antibodies (dilution 1:1000) for: p62 (#A7758), LC3BI/II (#A7198), p21 (#A19094), and phospho-PI3K (#AP0854), all purchased from Abclonal; Bcl-2 (#ab7973) and β-actin (#ab3280), both purchased from Abcam; phospho-mTOR (Ser2448) (#5536), phospho-AKT (Ser473) (#2983), PI3K (#42925), AKT (#92725) and mTOR (#2983), all purchased from Cell Signaling Technology, and p53 (#10442-1-AP, from Proteintech, IL, USA). Then, membranes were incubated with secondary antibodies HRP-conjugated goat anti-rabbit IgG (1:2,000, #111035003, Jackson Immuno Research Inc., West Grove, PA, USA).

### 2.14 Xenograft model

KKU-100 cells (1 × 10^6^ cells) were injected subcutaneously into female knockout BALB/c Rag-2.Jak3 double-knockout mice.^28^ After 21 days, mice were treated with either DMSO or 50 mg/kg·Bw of CBD (n = 5) daily for 4 days. Tumor sizes were measured at days 0, 3 and 5 following this, and the tumor volume was calculated using the formula: tumor volume = (length × width^2^)/2.

### 2.15 Immunohistochemistry study

Tumor tissues from mice were immunohistochemically analyzed by antigen retrieval with citrate buffer and Tris-EDTA buffer, blocking with 5 % FBS, and incubation with anti-proliferating cell nuclear antigen (PCNA) antibody (1:750, #Ab2426, Abcam), p21 antibody (1:50, #A19094, Abclonal), and GBL1 antibody (1:40, #A1863, Abclonal) overnight. The signal was detected with diaminobenzidine substrate and counterstained with Mayer's hematoxylin.^27^ Brown staining indicated positive cells. Ten fields (20 × magnification) were analyzed using ImageJ software.

### 2.16 Statistical analysis

The experiments were conducted in biological triplicate with results presented as mean ± SD. Statistical comparisons involved one-way ANOVA followed by Tukey's test for multiple group comparisons and Student's t-test for *in vivo* investigations with two cohorts.^29^ A significance threshold of *p* < 0.05 was used. All analyses were performed using GraphPad Prism 9.0 for Mac.

## 3 Results

### 3.1 CBD treatment suppresses CCA cell proliferation, induces cell cycle arrest and inhibits colony formation

**Fig. 1:**
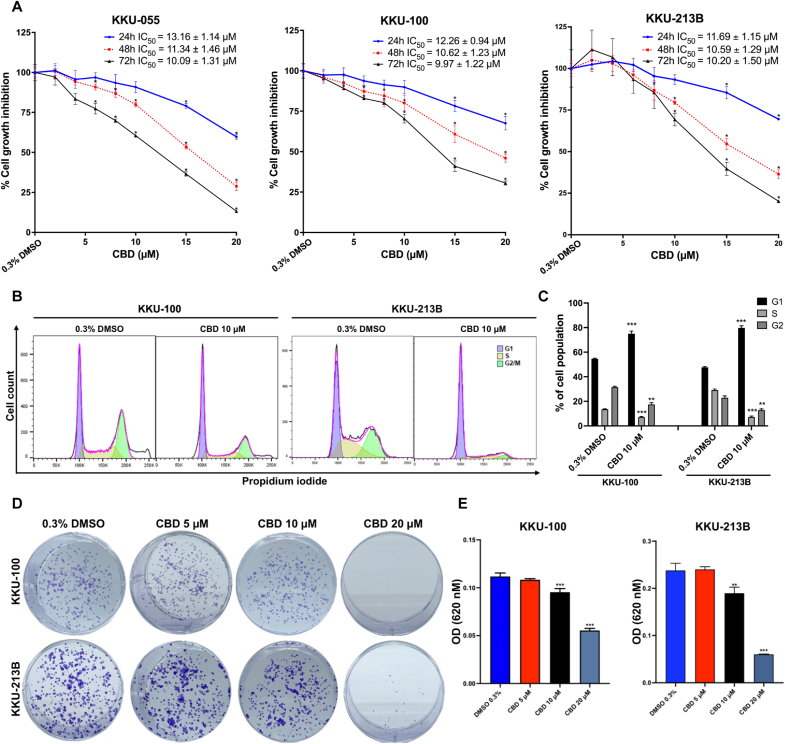
CBD treatment suppresses CCA cell proliferation, induces cell cycle arrest and inhibits colony formation. (A) The anti-proliferative activity of CBD against three CCA cell lines was evaluated using the SRB assay, with three biological replicates. (B) Flow-cytometric analysis of two CCA cell lines treated with 10 μM CBD indicated cell cycle arrest after 48 h. (C) Cell population percentages (n = 3) were calculated, and (D) clonogenic assay illustrated with representative images. (E) Absorbance values of cancer colonies at 620 nm were plotted in a bar chart (n = 3). All experiments were conducted with three biological and technical replicates. Data are presented as mean ± SD; significance levels denoted as * *p* < 0.05, **p < 0.01, and ***p < 0.0001 compared to the control group (0.3 % DMSO).

The SRB assay showed that CCA cell proliferation in all three cell lines decreased following CBD treatment in a dose- and time-dependentmanners (Fig. 1A). The half-maximal inhibitory concentration (IC_50_) values at 48 h of treatment were 11.34 ± 1.46, 10.62 ± 1.23, and 10.59 ± 1.29 μM for KKU-055, KKU-100 and KKU-213B, respectively. After 48 h of treatment, there were no differences in the IC_50_ values among the three distinct CCA cell lines. Cell line KKU-055 was not used in the remaining investigations reported below. While, IC_50_ for MMNK 1 was 12.73 ± 1.09, indicating that CBD has low toxic effect (Supplementary figure 1).

To further investigate the inhibitory effects of CBD on CCA cell proliferation, KKU-100 and KKU-213B cells were treated with 10 μM CBD for 48 h. Flow cytometric analysis revealed a significant increase in the proportion of KKU-100 and KKU-213B cells in the G1 phase (KKU-100; *p* < 0.001*,* KKU-213B; *p* < 0.001) after treatment with 10 μM CBD compared to the 0.3 % DMSO treatment (Fig. 1B and C).

To investigate the inhibitory effect of CBD on the proliferative capacity of a single CCA cell, a clonogenic assay was performed (Fig. 1D). CBD treatment inhibited colony formation in both CCA cell lines (KKU-100 and KKU-213B) in a dose-dependent manner. Treatment with CBD at 10 and 20 μM significantly inhibited colony formation of KKU-100 cells (*p* < 0.001 and *p* < 0.001, respectively) and KKU-213 (*p* < 0.01 and < 0.001, respectively) compared to vehicle control (0.3 % DMSO treatment) (Fig. 1E). Notably, 20 μM CBD exhibited greater efficacy than 10 μM CBD in inhibiting colony formation in both cell lines (Fig. 1D and E).

### 3.2 CBD induces lipid droplet and vesicle formation in CCA cells

**Fig. 2:**
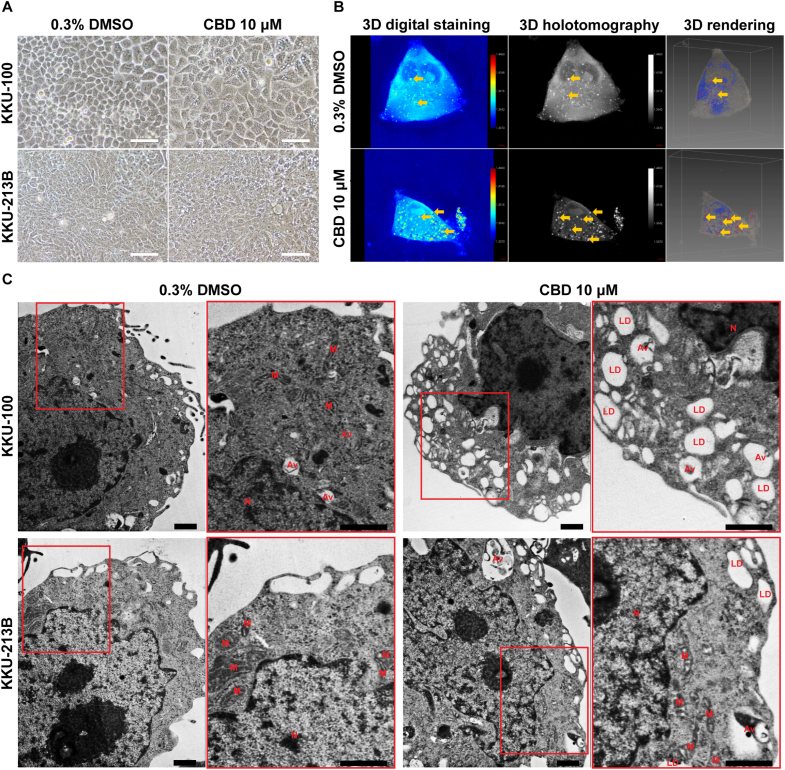
CBD induces lipid droplet and vesicle formation in CCA cells. (A) Representative images of the lipid droplet/vesicle formation in CCA cells induced by 10 μM CBD after 48 h, photographed under an inverted light microscope with a 0.40 mm scale bar. (B) The 3D rendering, 3D holotomography image, and 3D digital staining. The yellow arrows indicate intracellular fat droplets (high refractive index, RI). (C) TEM images were utilized to examine the effects of 10 μM CBD treatment for 48 h on the ultrastructure of CCA cells. Scale bar in the images represents 800 nm (N = Nucleus, M = Mitochondria, AV = Autophagosome (double membranes) and LD = Lipid droplet (single membrane).

Following a 48-h treatment with 10 μM CBD, KKU-100 CCA cells enlarged and contained various vesicles in the cytoplasm when observed using an inverted light microscope and 3D holotomography (Fig. 2A and B). This was confirmed by the observations obtained from TEM, which showed extensive cytoplasmic vacuolation, nucleolus enlargement, and the presence of autophagosomes (Fig. 2C). In addition, both CCA cell lines exhibited the presence of single- and double-membrane vacuoles.

### 3.3 CBD increases mitochondrial ROS, changes mitochondrial membrane potential (MMP; ΔΨm) and increases ROS production in CCA cells

**Fig. 3:**
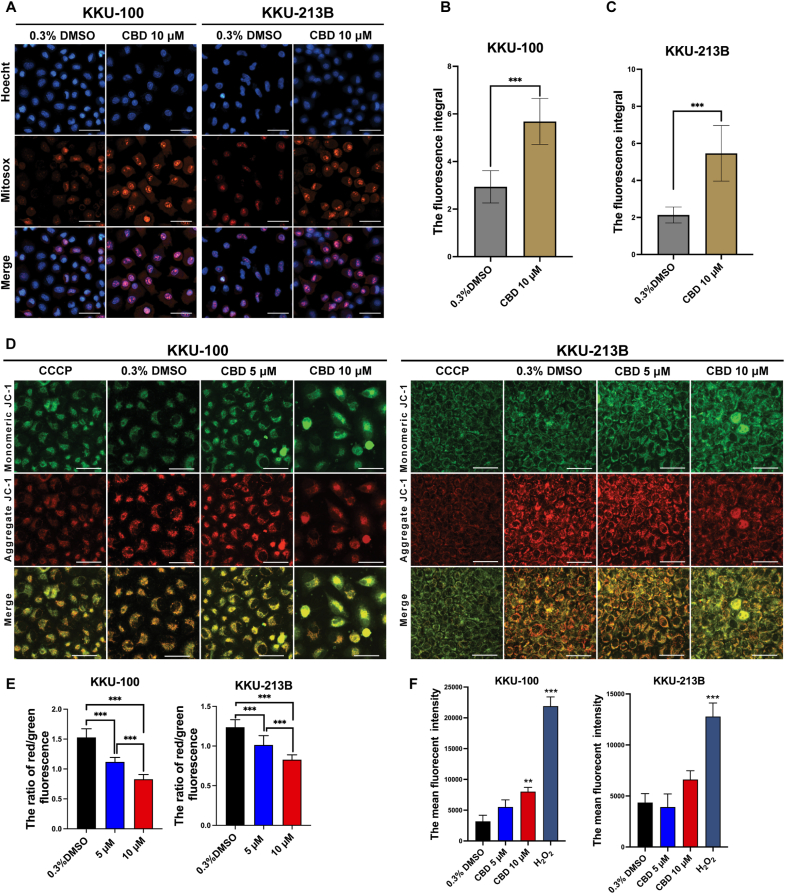
The effect of CBD on mitochondrial ROS, changes in mitochondrial membrane potential (MMP; ΔΨm) and ROS production in CCA cells. (A) Superoxide levels, measured with MitoSOX in CCA cells treated with 10 μM CBD compared to the vehicle control over 48 h. Cellular imaging included Hoechst 33,342 for nuclear staining, MitoSOXstaining, and merged images. (B–C) Bar charts represented quantification of mitochondrial superoxide levels through fluorescence intensity. (D) Mitochondrial membrane potential in CCA cells was assessed using JC-1 staining after 48 h of CBD treatment. Images were acquired at 20× magnification with a 50 μm scale bar. Quantitative analysis was done using Image J software, with data collected from ten fields per condition, replicated in triplicate. (E) A bar chart depicting analysis of the red/green ratio. (F) Flow cytometry analysis of CCA cells stained with DCFH-DA showing mean fluorescence intensity for ROS detection. Data are presented as mean ± SD (n = 3); *p < 0.05, **p < 0.01, and ***p < 0.0001.

Fig. 3 demonstrates the effect of CBD on mitochondrial ROS, changes MMP and increases ROS production in CCA cells. Induction of ROS generation in mitochondria was evaluated using MitoSOX Red as a fluorescent probe. Elevated orange-red fluorescence indicated increased mitochondrial ROS levels. Treatment with 10 μM CBD significantly boosted fluorescence intensity in KKU-100 and KKU-213B cell lines (*p* < 0.0001 in both cases) compared to vehicle control (Fig. 3A–C). Additionally, cells treated with vehicle control (0.3 % DMSO) exhibited a higher proportion of red fluorescence,indicating healthy mitochondria, whereas, cells treated with 50 μM CCCP or CBD displayed a higher proportion of green fluorescence,indicating mitochondrial damage, relative to red fluorescence (Fig. 3D). In CBD treatment, the red/green fluorescence ratio of KKU-100 cells significantly decreased to 1.12 ± 0.07 (*p* < 0.001) and 0.83 ± 0.08 (*p* < 0.001) with CBD at concentrations of 5 and 10 μM, respectively, when compared to vehicle control (1.53 ± 0.14). Similarly, the red/green ratio of KKU-213B cells was significantly lower at 1.01 ± 0.12 (*p* < 0.001) and 0.83 ± 0.06 (*p* < 0.001) by CBD treatment at concentrations of 5 and 10 μM, respectively, when compared to vehicle control (1.24 ± 0.10) (Fig. 3E).

Flow cytometry revealed that KKU-100 CCA cells displayed a slight increase in fluorescence intensity when treated with 5 μM CBD and a significant rise (*p* < 0.01) with 10 μM CBD after 12 h. This did not occur in KKU-213B cells. As expected, both CCA cell lines gave the highest fluorescence intensity with 1 mM H_2_O_2_, the positive ROS control, after 3 h (Fig. 3F).

### 3.4 CBD induces autophagy in CCA cells

**Fig. 4:**
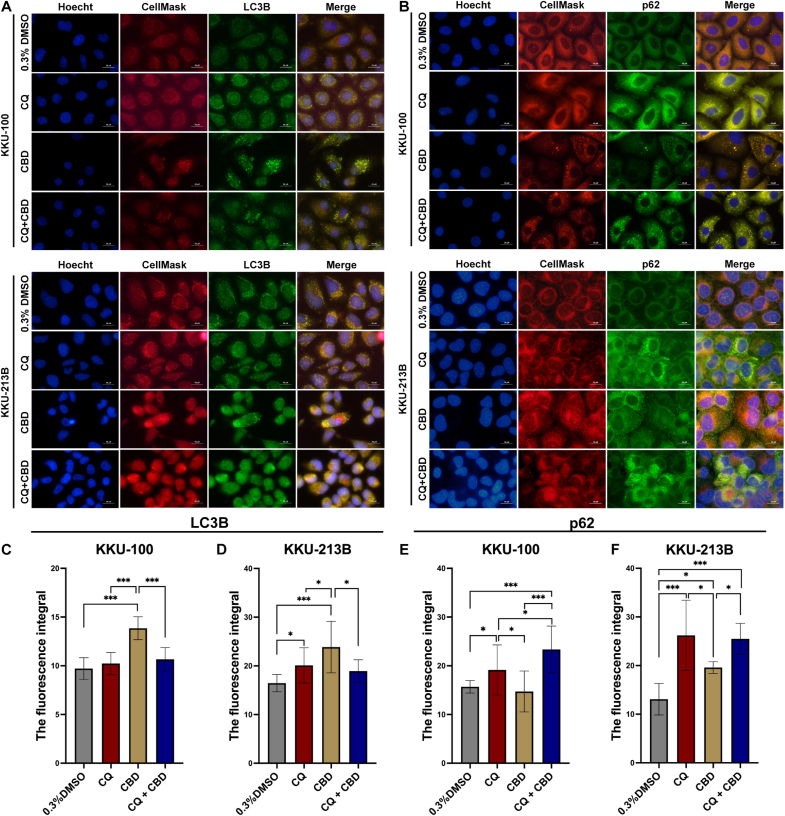
CBD induces autophagy in CCA cells. (A–B) Representative immunofluorescent images for autophagy markers. Each cell image series features four columns: nuclear staining with Hoechst, Cellmask staining for the cell membrane, green fluorescence indicating LC3 or p62 expression, and merged images. Images were captured at 100× magnification with a 50 μm scale bar. (C–F) Bar charts depicting fluorescent integral values, normalized to areas using ImageJ software. Statistical significance is indicated as; *p < 0.05, ** *p* < 0.01, and ***p < 0.0001.

We further examined the impact of co-administration of CBD and CQ, an autophagy inhibitor, on autophagy pathways through IFA (Fig. 4) and Western blot analysis. IFA revealed increased LC3 fluorescence following CBD and CQ co-administration compared to 0.3 % DMSO control (*p* < 0.0001) (Fig. 4A and 4C-D). CBD treatment also altered p62 fluorescence, with significant effects in KKU-213B (*p* = 0.025) but not KKU-100 (*p* = 0.9023) (Fig. 4B and 4E-F). In contrast, CBD treatment led to a significant reduction in fluorescence intensity of p62 compared to CQ alone or the combination of CQ with CBD.

**Fig. 5:**
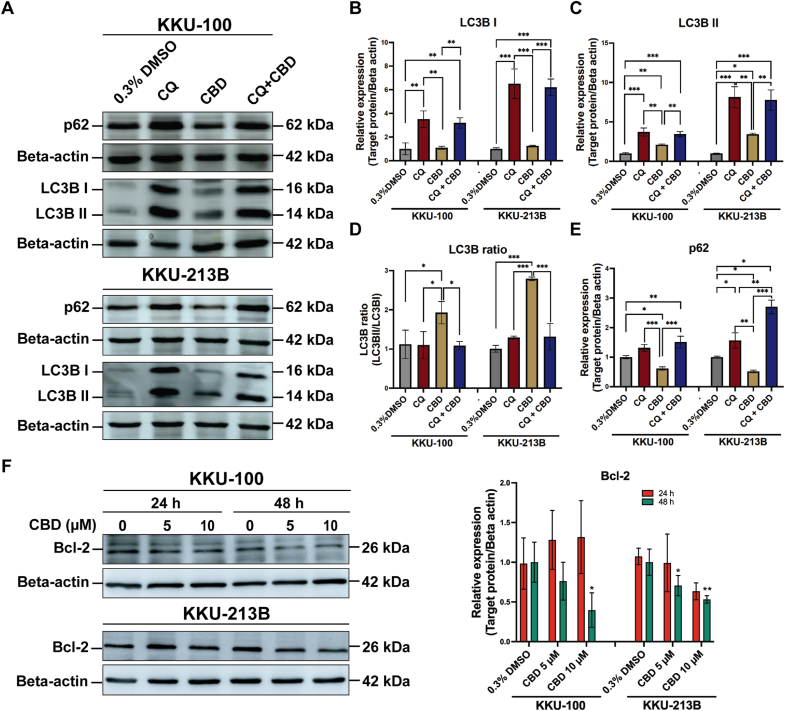
Effects of CBD on the expression of protein markers associated with autophagy and anti-autophagy in CCA cells. The relative fold changes of protein expression are shown as bar graphs based on the intensity of the protein band compared with β-actin as a loading control (**A - G**). *p < 0.05, **p < 0.01 and ***p < 0.0001.

We further confirmed this by Western blot analysis (Fig. 5). CQ alone or with CBD significantly increased LC3B–I and II expression compared to 0.3 % DMSO or CBD treatment alone. CBD at 10 μM notably elevated LC3B-II expression in both cell lines compared to 0.3 % DMSO alone (KKU-100: *p* = 0.011, KKU-213B: *p* = 0.049). CBD treatment exhibited the most pronounced LC3B-II/I ratio (*p* < 0.0001) (Fig. 5A–D). A significantly decreased expression of p62 levels was observed in CCA cell lines with CBD treatment compared to 0.3 % DMSO or CQ alone (Fig. 5A and E). Additionally, CBD treatment reduced Bcl-2 expression levels in a dose-dependent manner. Significant reductions in Bcl-2 expression were observed at various CBD concentrations (KKU-100: 10 μM, *p* = 0.036; KKU-213B: 5 μM, *p* = 0.027; 10 μM, *p* = 0.007) relative to vehicle control (Fig. 5F).

### 3.5 CBD suppresses the expression of the PI3K/Akt/mTOR pathway

**Fig. 6:**
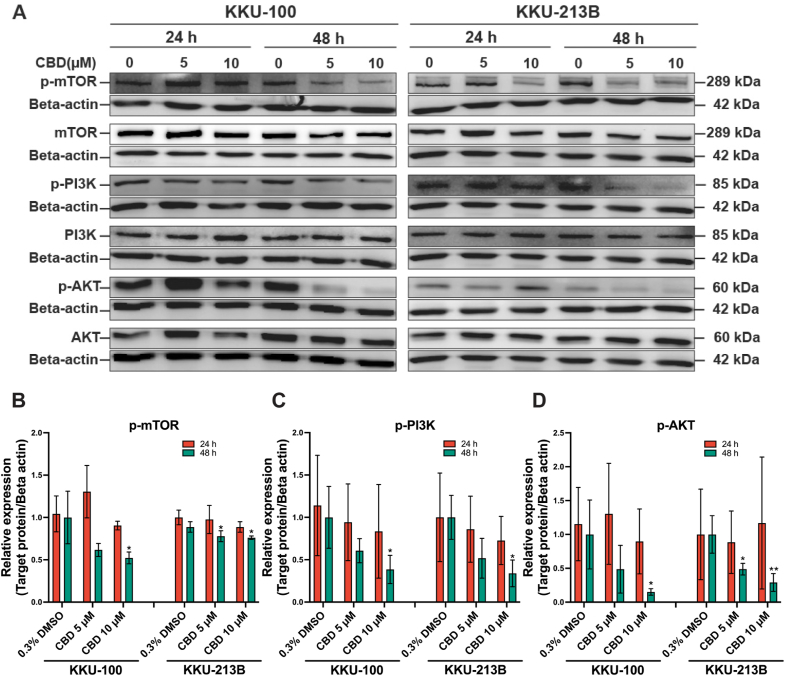
CBD suppresses protein expression in the PI3K/AKT/mTOR pathway in CCA cells. (A) Western blot analysis of PI3K/AKT/mTOR pathway components in CCA cells treated with CBD at 5 and 10 μM. The relative intensities of protein bands were quantified in relation to beta-actin, which served as a loading control on each membrane. (B–D) The relative fold changes in protein expressions are presented as bar graphs. *p < 0.05, **p < 0.01, and ***p < 0.001. The data were derived from three independent experiments.

To investigate the impact of CBD on the PI3K/Akt/mTOR pathway, Western blot analysis was conducted (Fig. 6). The basal expression of PI3K, Akt and mTOR in KKU-100 and KKU-213B cells did not differ between the control and CBD treatment groups. However, in KKU-100 cells treated with 5 and 10 μM CBD, there was a reduction in the expression of phosphorylated PI3K (*p*-PI3K), phosphorylated Atk (*p*-Atk) and phosphorylated mTOR (*p*-mTOR) at 24 h compared to the control group. Interestingly, relative to controls, KKU-100 cells treated with 10 μM CBD exhibited a significant decrease in the expression of *p*-PI3K (*p* = 0.039*)*, *p*-AKT (*p* = 0.048), and *p*-mTOR (*p* = 0.037) at 48 h. Likewise, KKU-213B cells treated with CBD 10 μM showed a slight decrease in the expression of *p*-PI3K, *p*-AKT, and *p*-mTOR at 24 h, but a significant decrease in these proteins was observed at 48 h (*p* = 0.019, 0.006 and 0.030, respectively; Fig. 6A and D).

### 3.6 CBD promotes cellular senescence in CCA cells

**Fig. 7:**
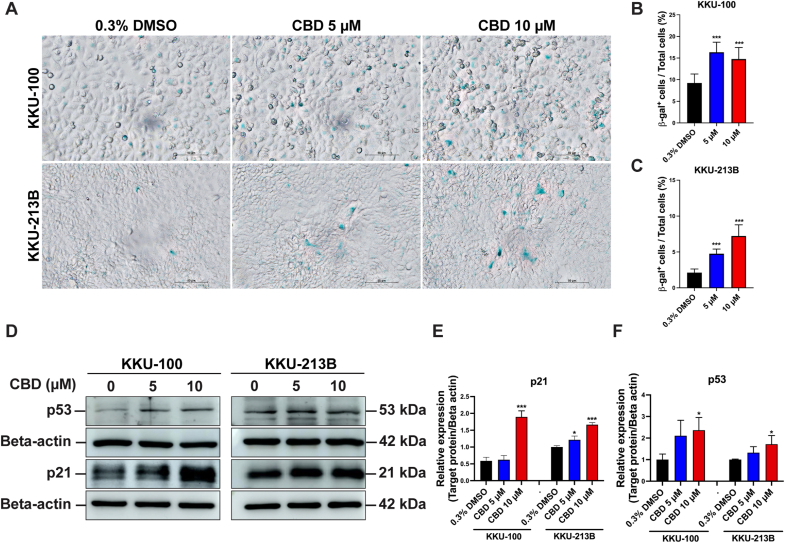
CBD induced cellular senescence in CCA cell lines. (A) Senescence-associated β galactosidase (SA-β-Gal) staining in either 0.3 % DMSO- or 10 μM CBD-treated CCA cells for 48 h. Senescent cells stained blue. (B–C) Bar graphs display the mean ± SD of the percentage of SA-β-Gal positive cells divided by the total number of cells. (D) Western blot analysis of p53 and p21 proteins in CCA cell lines treated with solvent control (0.3 % DMSO) and CBD (5 and 10 μM) for 24 h. (E–F) The relative fold change of protein expression shown as a bar graph based on the intensity of the protein band relative to β-actin, a loading control. *p < 0.05, **p < 0.01 and ***p < 0.0001 compared to the control.

To evaluate the effect of CBD on induction of cellular senescence in CCA cell lines, SA-β-gal staining was performed. SA-β-gal appeared deep blue as shown in Fig. 7A. Cellular senescence significantly increased in KKU-100 and KKU-213B cells treated with 5 μM and 10 μM (*p* < 0.0001) compared to the vehicle control (Fig. 7B and C). To confirm cellular senescence, a Western blot analysis was performed to examine the cellular senescence markers p21 and p53 (Fig. 7D). CBD treatment (10 μM) of KKU-100 and KKU-213B for 24 h elicited a significant upregulation in the expression of p21 (KKU-100: *p* < 0.0001) when contrasted with treatment involving 0.3 % DMSO (Fig. 7E). In addition, p53 expression was found to be significantly elevated in the groups treated with 10 μM CBD (KKU-100: *p* = 0.042, KKU-213B: *p* = 0.035; Fig. 7D and F), confirming the activation of cell senescence by CBD in CCA cells.

### *3.7* CBD suppresses CCA cell proliferation and promotes cellular senescence *in vivo*

**Fig. 8:**
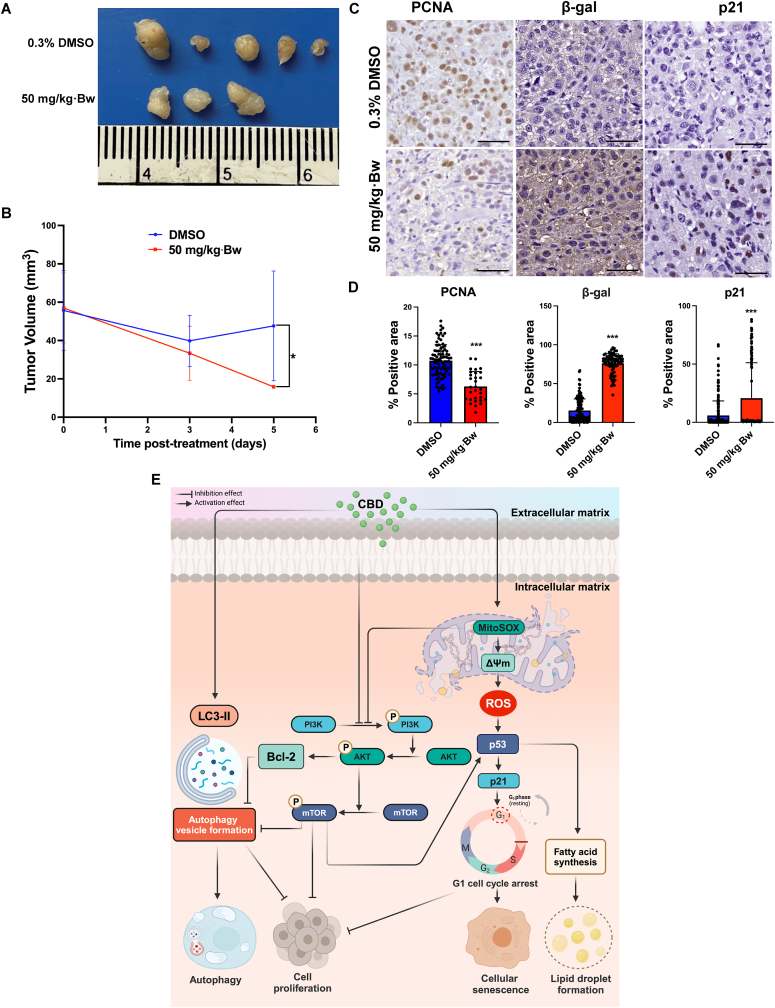
The anti-tumor effects of CBD in a CCA xenograft mice model. KKU-100 cells (1 × 10^6^) were subcutaneously injected into the animal. Twenty-one days later, mice were treated by intraperitoneal injections with DMSO or CBD (50 mg/kg) each day for 4 days. (**A**) Representative tumor masses removed from mice. (**B**) Tumor volumes (mm^3^) were measured on days 0, 3 and 5 post-treatment. (**C**) Representative immunohistochemistry images of tumor-mass sections stained for PCNA, a marker of cell proliferation, β-Gal and p21 a recognized marker of cellular senescence. (**D)** The grading scores attributed to each marker within the tumor mass tissues denoted as the mean +SD. *p < 0.05, **p < 0.01, and ***p < 0.0001. (**E**) Postulated mechanism of CBD against CCA via the PI3K/AKT/mTOR pathway. This figure was created by BioRender.com (license no. XG26NE9GPA).

The tumor volume of xenograft mice treated with 50 mg/kg·Bw was significantly lower upon evaluation on day 5 after treatment (*p* = 0.0357) when compared to vehicle control treatment (Fig. 8A and B). Additionally, an immunohistochemistry study was performed to confirm the effect of CBD on the suppression of CCA proliferation and induction of cellular senescence at the molecular level (Fig. 8C). The expression of PCNA, β-gal and p21 were assessed and its area and intensity were measured using ImageJ software (Fig. 8D). The expression of PCNA in the 50 mg/kg·Bw group was significantly reduced (*p* < 0.0001) relative to the vehicle control group. Immunoreactivity indicated a significant increase in β-gal levels in mice treated with 50 mg/kg·Bw of CBD (*p* < 0.0001). Consistently, a noteworthy elevation in p21 expression was observed in tumor tissues by CBD treatment at the same dosage (*p* < 0.0001).

## 4 Discussion

Although CBD has shown anti-tumor activity in various solid tumors, including CCA, its mechanism of action remains poorly understood.^30^^,^^31^ This study provides, for the first time, an insight into the *anti*-CCA mechanism of CBD and highlights its effect *in vitro* and *in vivo* via multiple mechanisms. Specifically, CBD was found to inhibit cell proliferation, colony formation, induce micro- and ultrastructural changes, disrupt mitochondrial membrane potential and increase mitochondrial ROS generation, and increase ROS production. In addition, CBD induced autophagy by suppression of the PI3K/AKT/mTOR pathway, activated cell cycle arrest and induced cellular senescence in CCA cells. These results suggest that CBD holds potential as an effective treatment for CCA. The proposed *anti*-CCA mechanism of CBD is depicted in Fig. 8E.

Various herbal agents, including CBD, have shown promise for the treatment of CCA.^32^ Viereckl and colleagues have reported high efficacy of CBD within a dosage range of 50–100 μM.^31^ In this study, we determined lower IC_50_ values for CBD (10.59–11.43 μM), suggesting heightened sensitivity of CCA to CBD in the context of *O. viverrini* infection. Our study supports this concept by demonstrating the suppression of tumor cell growth by CBD in both *in vitro* and *in vivo* models. Specifically, CBD administration in animal models resulted in a reduction in tumor cell division by downregulating the expression of PCNA, highlighting its potential clinical applications. The anti-CCA activity of CBD can be attributed, at least in part, to its ability to inhibit cancer cell division and prevent colony formation, providing insight into the underlying mechanism of action.

The modulation of cell cycle regulation by phytochemicals and natural products represents an important strategy to combat tumor cell proliferation, metastasis, and recurrence in various cancers.^33^^,^^34^ Among these agents, CBD has emerged as a notable candidate known for its ability to arrest the cell cycle by upregulating the expression of p53 and p21 in solid and liquid tumors.^34^, ^35^, ^36^, ^37^ Consistent with previous research, our study showed that CBD treatment induces G1 cell cycle arrest in CCA cells, mediated in part by upregulation of p53 and p21 expression. This finding is consistent with similar observations in gastric cancer,^34^ chronic myeloid leukemia,^37^ and ovarian cancer,^36^ highlighting the potential of CBD as a promising therapeutic agent for targeting abnormal cell cycle progression in cancer treatment.

Herbal medicines, including CBD, are known for their ability to affect cancer cell morphology and to induce cell death^30^ by induction of mitotically arrested and apoptotic cells, leading to lipid accumulation.^38^ In our study, CBD treatment induced the formation of lipid droplets and vesicles, altering the characteristics of CCA cells. Relevantly, CBD treatment increased lipid accumulation in human mesenchymal stem cells,^39^ and induced vesicle formation in human lung cancer cells,^40^ suggesting CBD treatment may have an effect due to its role in cell cycle arrest, apoptosis, and cell death, possibly through activation of adipogenesis-related metabolic pathways via PPARγ.^40^

The use of natural products, such as CBD, to target autophagy has shown promise for the prevention and treatment of various types of cancer^41^ by decreasing the activity of the PI3K/AKT/mTOR pathway.^30^ As we found, CBD inhibited CCA growth by reducing phosphorylation of the PI3K/AKT/mTOR pathway in relation to a decrease in the expression of Bcl-2, a protein involved in cell survival, and an increase in autophagy. Similar effects of CBD on autophagy have been observed in breast cancer^30^ and bladder cancer.^42^ Inhibition of autophagosome formation by CQ causes accumulation of LC3-II and p62 while an increase in LC3BII levels and a decrease in p62 levels were demonstrated following CBD treatment in CCA cells. The results suggested that CBD induces autophagy via the conversion of LC3-I to LC3-II, leading to an increase in LC3-II, while the amount of p62 would be decreased by lysosomal degradation. These findings are consistent with prior studies that have utilized plant-based compounds to induce autophagy in CCA.^27^ Essentially, our results indicate that CBD influences the signaling pathways of LC3II and p62, while simultaneously reducing the expression of certain proteins such as *p*-PI3K, *p*-AKT, *p*-mTOR, and Bcl-2 in CCA cells, suggesting that CBD has potential therapeutic application in various cancer types, including CCA.

In addition to modulating autophagy, targeting cellular senescence through interruption of ROS production is a promising strategy for cancer treatment. The effect of CBD on ROS production was observed in two CCA cell lines, KKU-100 and KKU-213B. While CBD increased ROS levels in KKU-100 cells, it did not have the same effect in KKU-213B cells, indicating possible differences in glutathione redox status between these cell types.^43^ Our findings revealed that CBD treatment not only induced the production of ROS but also led to an increase in MitoSOX, indicating enhanced mitochondrial ROS generation. CBD treatment stimulated the expression of p53 and its downstream effector, p21, both of which are pivotal regulators of cellular senescence.^44^ Moreover, wide-type p53 inhibits the fatty acid synthesis and lipid accumulation, whereas mutant p53 that found in cancer cells inhibits AMPKα and activates SREBPs, resulting in enhances fatty acid biosynthesis and lipid droplet formations.^45^ Consistent with our observations, the induction of cellular senescence in HepG2 cells using the plant derivative 1,8-cineole increased ROS production, inhibited AKT phosphorylation, and altered mitochondrial membrane potential and induced cellular senescence^46^ in a manner similar to CBD. Notably, our *in vitro* findings were further supported by tumor xenograft tissues from mice treated with CBD, which revealed elevated levels of p21 and β-gal, indicative of increased cellular senescence. Hence, CBD may function as a therapeutic agent for CCA treatment by targeting mitochondrial ROS and cellular senescence pathways.

In addition, plant-based chemicals such as resveratrol promote autophagy-mediated cancer cell dormancy in ovarian cancer^47^ and in CCA.^48^ Likewise, our CBD treatment in CCA alters autophagic markers, mediates cell cycle arrest, induces cellular senescence, and inhibits cell proliferation. CBD-induced autophagy may foster cell dormancy, typified by reduced metabolic activity and proliferation, providing cancer cell resilience under adverse conditions.^47^

It is important to acknowledge certain limitations regarding the anti-tumor effects of CBD observed in our study. We used a xenograft model of CCA in immunocompromised mice, a model which may not reflect the complex interaction between CBD and the tumor environment in humans, suggesting that further studies on an orthotopic xenograft model may better mimic than subcutaneous xenograft.^49^

## 5 Conclusions

The study reported here has shown that CBD has a significant anti-tumor effect on CCA cells through various mechanisms, including the inhibition of cell proliferation both *in vitro* and *in vivo*, the reduction of colony formation ability and the induction of multiple cellular processes, notably autophagy, cell cycle arrest, cellular senescence, mitochondrial dysfunction, lipid droplet formation, and ROS overproduction. The significant findings from our study strongly suggest that CBD, through its targeting of the PI3K/AKT/mTOR pathway, holds great promise as a therapeutic agent for treating CCA and potentially other cancers.

## CRediT authorship contribution statement

Thatsanapong Pongking: Conceptualization, Data curation, Formal analysis, Methodology, Roles/Writing - original draft., Kitti Intuyod, Phonpilas Thongpon and Raynoo Thanan: Conceptualization, Investigation, Data curation, Methodology, Resources, Validation, Writing - review & editing., Chutima Sitthirach, Suppakrit Kongsintaweesuk, and Sirinapha Klungsaeng: Data curation, Investigation, Methodology. Apisit Chaidee, Nuttanan Hongsrichan, and Kulthida Vaeteewoottacharn: Investigation, Methodology, Validation. Chadamas Sakonsinsiri and Somdej Kanokmedhakul: Resources, Validation. Porntip Pinlaor and Somchai Pinlaor: Funding acquisition, Project administration, Resources, Supervision, Validation, Visualization, Writing - review & editing. The final manuscript was reviewed and approved by all authors. All authors agree to be accountable for all aspects of work ensuring integrity and accuracy.

## Ethics approval

The protocol of study in CCA cell lines was approved by Khon Kaen University Ethics Committee for Human Research based on the declaration of Helsinki and the ICH good clinical practice guidelines (HE641530). Animal experiments were conducted following the Animal Ethics Committee of Khon Kaen University and the National Research Council of Thailand's guidelines (No. IACUC-KKU-138/64).

## Funding

This research has received funding support from the National Science Research and Innovation Fund (NSRF) via the Program Management Unit for Human Resources & Institutional Development, Research and Innovation [grant number B05F630053/5856] and the Basic Research Fund of Khon Kaen University under Cholangiocarcinoma Research Institute (CARI-BRF64-4). Thatsanapong Pongking and Porntip Pinlaor were supported by National Research Council of Thailand (NRCT) through NRCT5-RGJ63003-055.
