# Corrigendum to “Cannabidiol suppresses proliferation and induces cell death, autophagy and senescence in human cholangiocarcinoma cells via the PI3K/AKT/mTOR pathway” [J Tradition Complement Med 14(6) (2024) 622–634]

**DOI:** 10.1016/j.jtcme.2025.01.001

**Published:** 2025-01-02

**Authors:** Thatsanapong Pongking, Kitti Intuyod, Phonpilas Thongpon, Raynoo Thanan, Chutima Sitthirach, Apisit Chaidee, Suppakrit Kongsintaweesuk, Sirinapha Klungsaeng, Nuttanan Hongsrichan, Chadamas Sakonsinsiri, Kulthida Vaeteewoottacharn, Somdej Kanokmedhakul, Somchai Pinlaor, Porntip Pinlaor

**Affiliations:** a Biomedical Sciences Program, Graduate School, Khon Kaen University, Khon Kaen, 40002, Thailand; b Department of Pathology, Faculty of Medicine, Khon Kaen University, Khon Kaen, 40002, Thailand; c Department of Parasitology, Faculty of Medicine, Khon Kaen University, Khon Kaen, 40002, Thailand; d Department of Biochemistry, Faculty of Medicine, Khon Kaen University, Khon Kaen, 40002, Thailand; e Centre for Research and Development of Medical Diagnostic Laboratories, Faculty of Associated Medical Sciences, Khon Kaen University, Khon Kaen, 40002, Thailand; f Department of Chemistry, Faculty of Science, Khon Kaen University, Khon Kaen, 40002, Thailand; g Cholangiocarcinoma Research Institute, Faculty of Medicine, Khon Kaen University, Khon Kaen, 40002, Thailand

The authors regret that the manuscript needs small corrections which were inadvertently missed.

It has come to our attention that the animal ethics number in the above-mentioned article was incorrectly stated as **No. IACUC-KKU-138/64 (former)**. The correct animal ethics number is **No. IACUC-KKU (C)- 92/66**.

The authors apologize for this oversight and any confusion it may have caused.

