# Supplementary material for: Cannabidiol suppresses proliferation and induces cell death, autophagy and senescence in human cholangiocarcinoma cells via the PI3K/AKT/mTOR pathway
Source: J Tradit Complement Med. 2024 Apr 17;14(6):622–34. doi: 10.1016/j.jtcme.2024.04.007 (PMC11752120; doi:10.1016/j.jtcme.2024.04.007)
Supplement: Multimedia component 3 [file mmc3.docx]

**Supplementary materials**


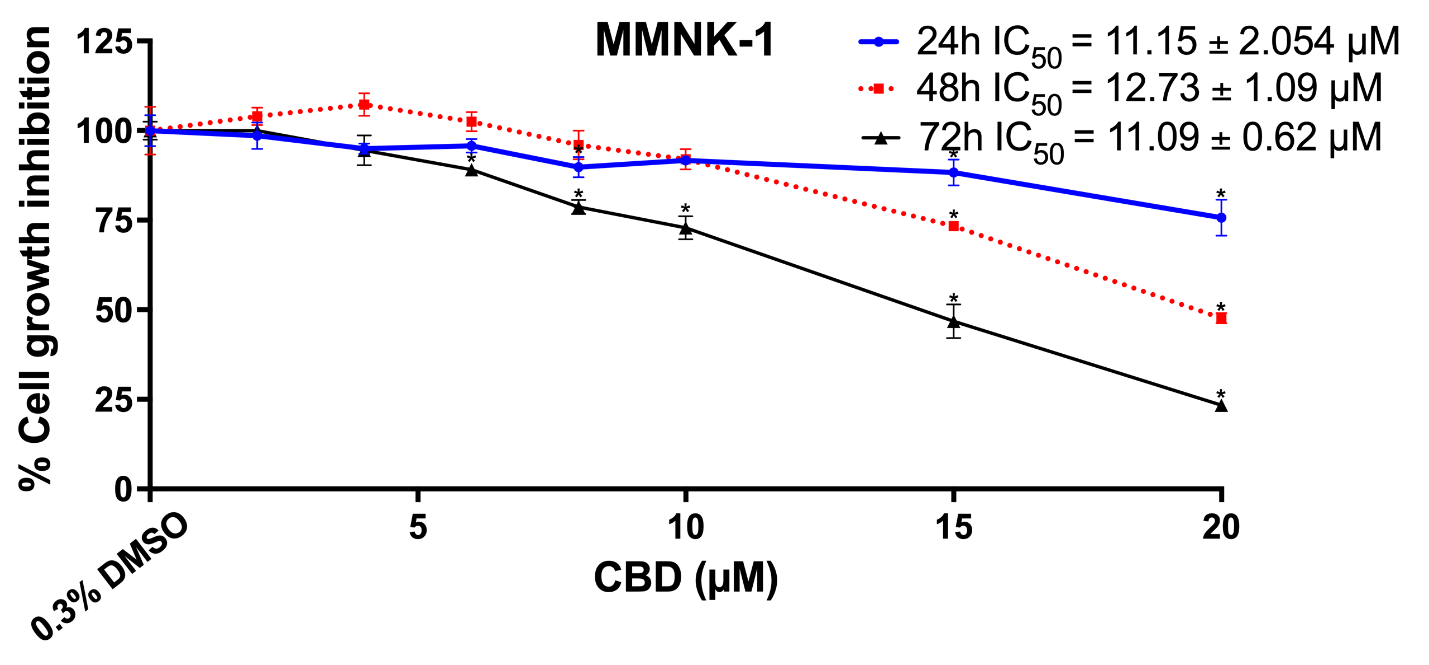


**Supplementary figure 1** The anti-proliferative activity of CBD against MMNK-1 cholangiocyte cell line (non cancerous) was evaluated using the SRB assay, with three biological replicates.
