# Supplementary material for: Cannabidiol suppresses proliferation and induces cell death, autophagy and senescence in human cholangiocarcinoma cells via the PI3K/AKT/mTOR pathway
Source: J Tradit Complement Med. 2024 Apr 17;14(6):622–34. doi: 10.1016/j.jtcme.2024.04.007 (PMC11752120; doi:10.1016/j.jtcme.2024.04.007)
Supplement: Multimedia component 4 [file mmc4.docx]

**Supplementary materials**

**Supplementary table 1** Original blotted membranes from KKU-213B and KKU-100 cell lines were treated with varying concentrations of Cannabidiol (CBD) at 5 and 10 µM for 24 and 48 hours. A control group treated with 0.3% DMSO served as the vehicle control to investigate the PI3K/Akt/mTOR signaling pathways and Bcl-2. In the western blot analysis, lane 1 represents the molecular weight marker, lanes 2 through 4 depict the CCA cell-line samples treated with 0.3% DMSO, CBD at 5 µM, and CBD at 10 µM for 24 h, respectively. Similarly, lanes 5 through 7 illustrate the CCA cell-line samples treated with 0.3% DMSO, CBD at 5 µM, and CBD at 10 µM for 48 h, respectively.

| Cell lines | Protein target | Original membranes of target protein | Beta-actin |
| --- | --- | --- | --- |
| KKU-213B | p-mTOR | 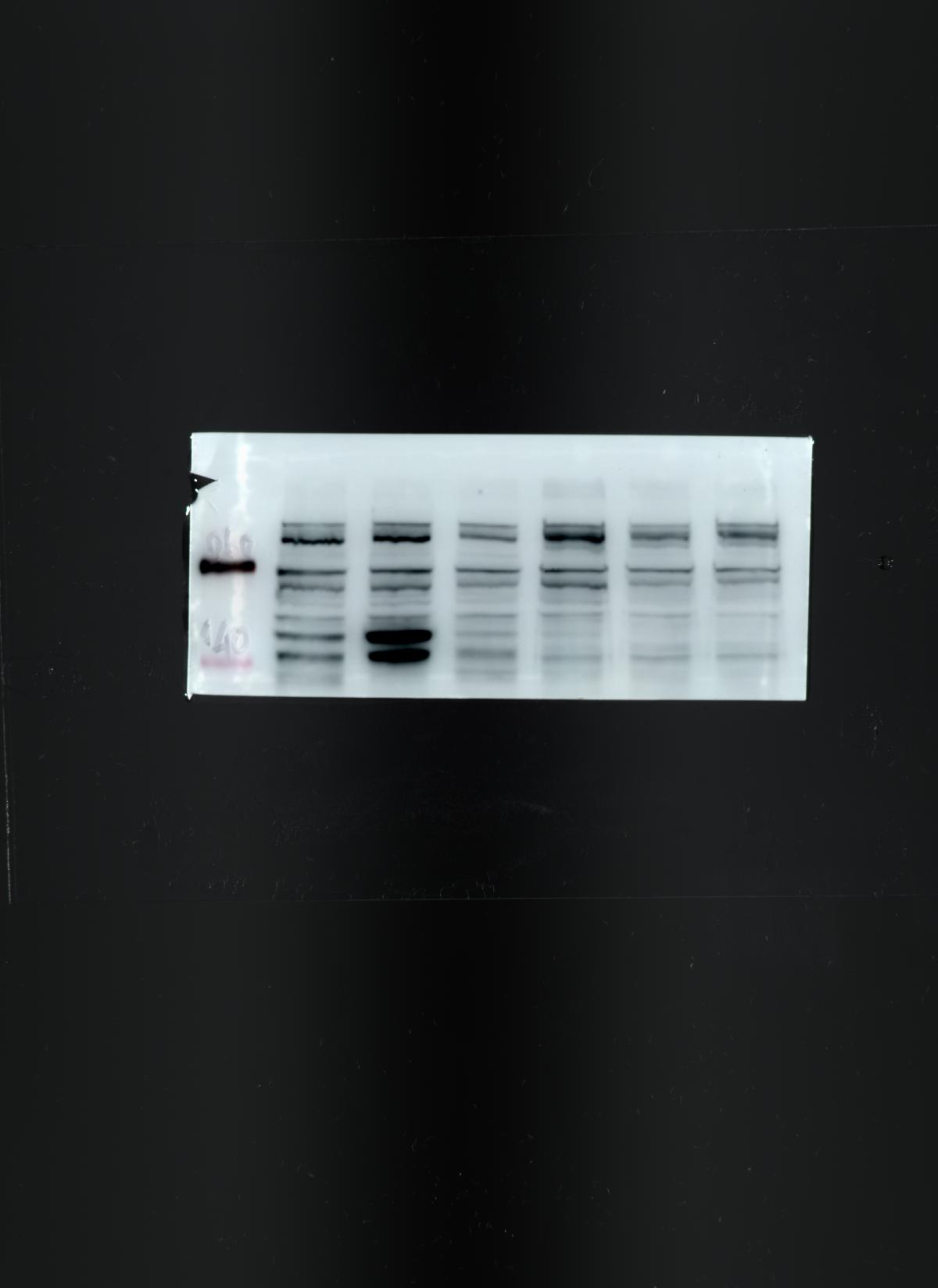 | 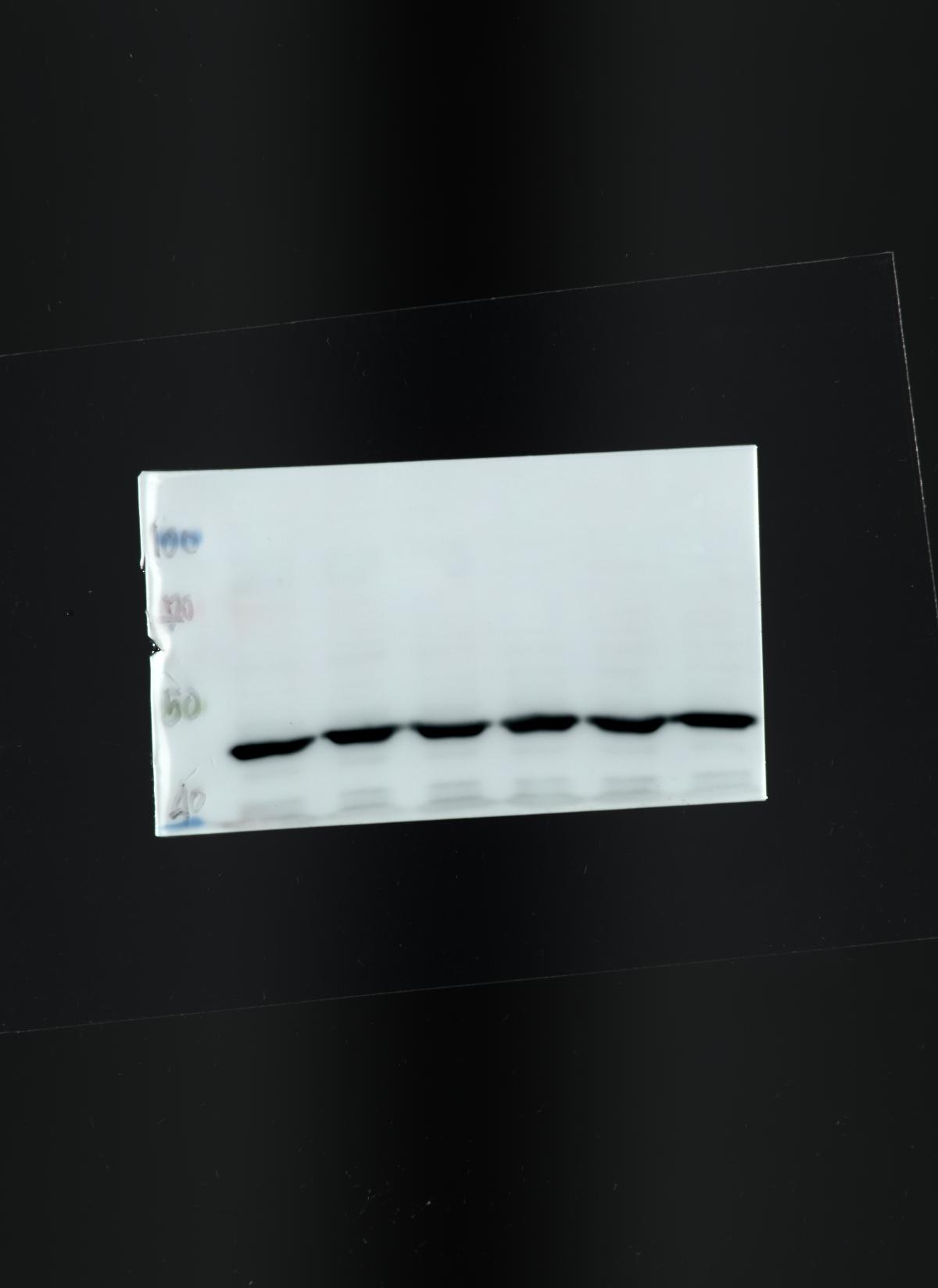 |
| KKU-213B | mTOR | 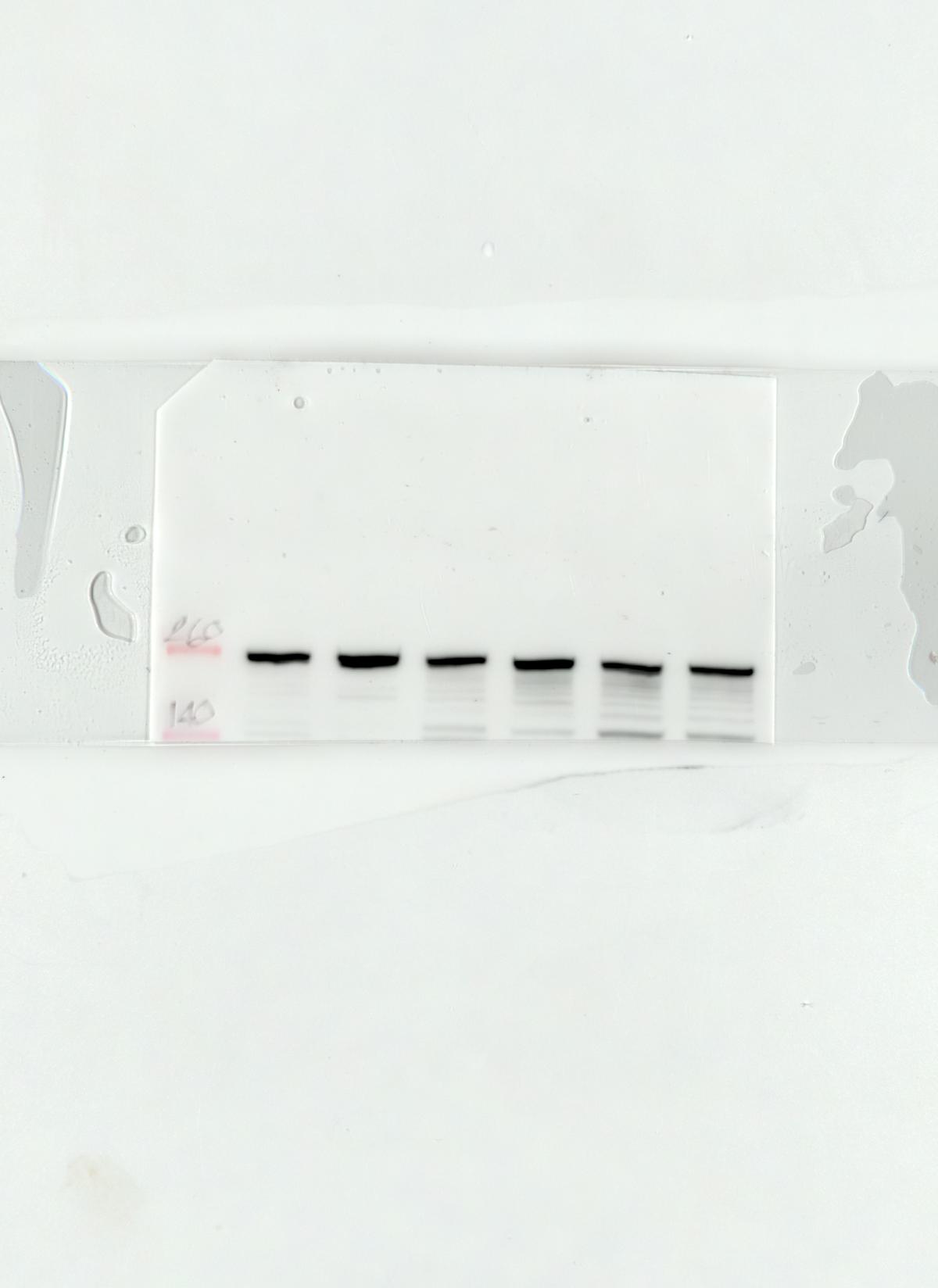 | 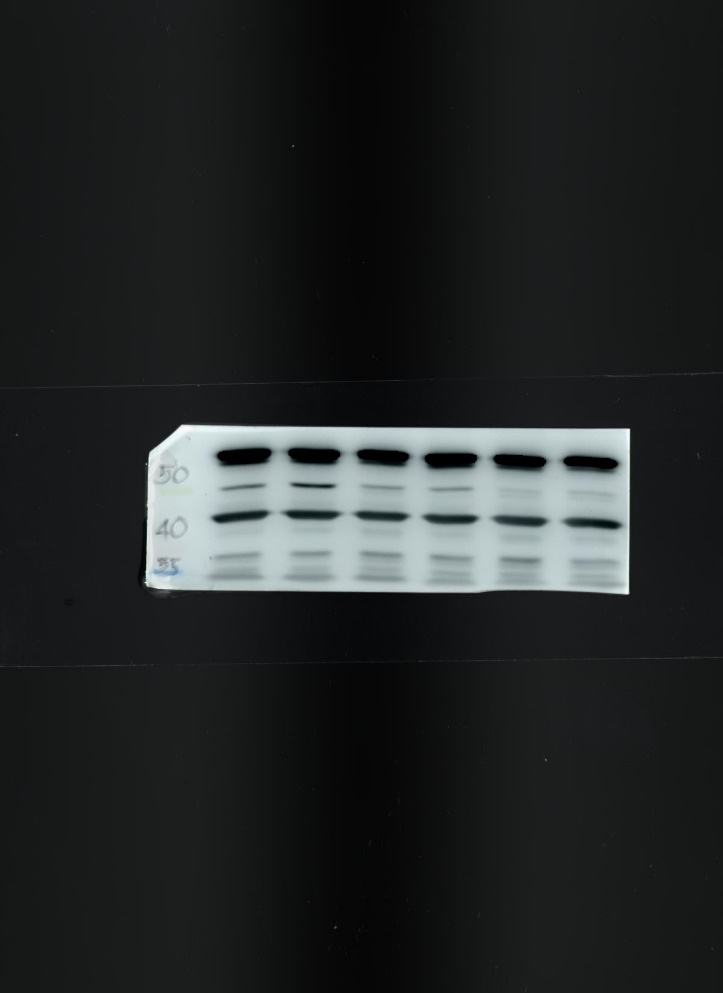 |
| KKU-213B | p-PI3K | 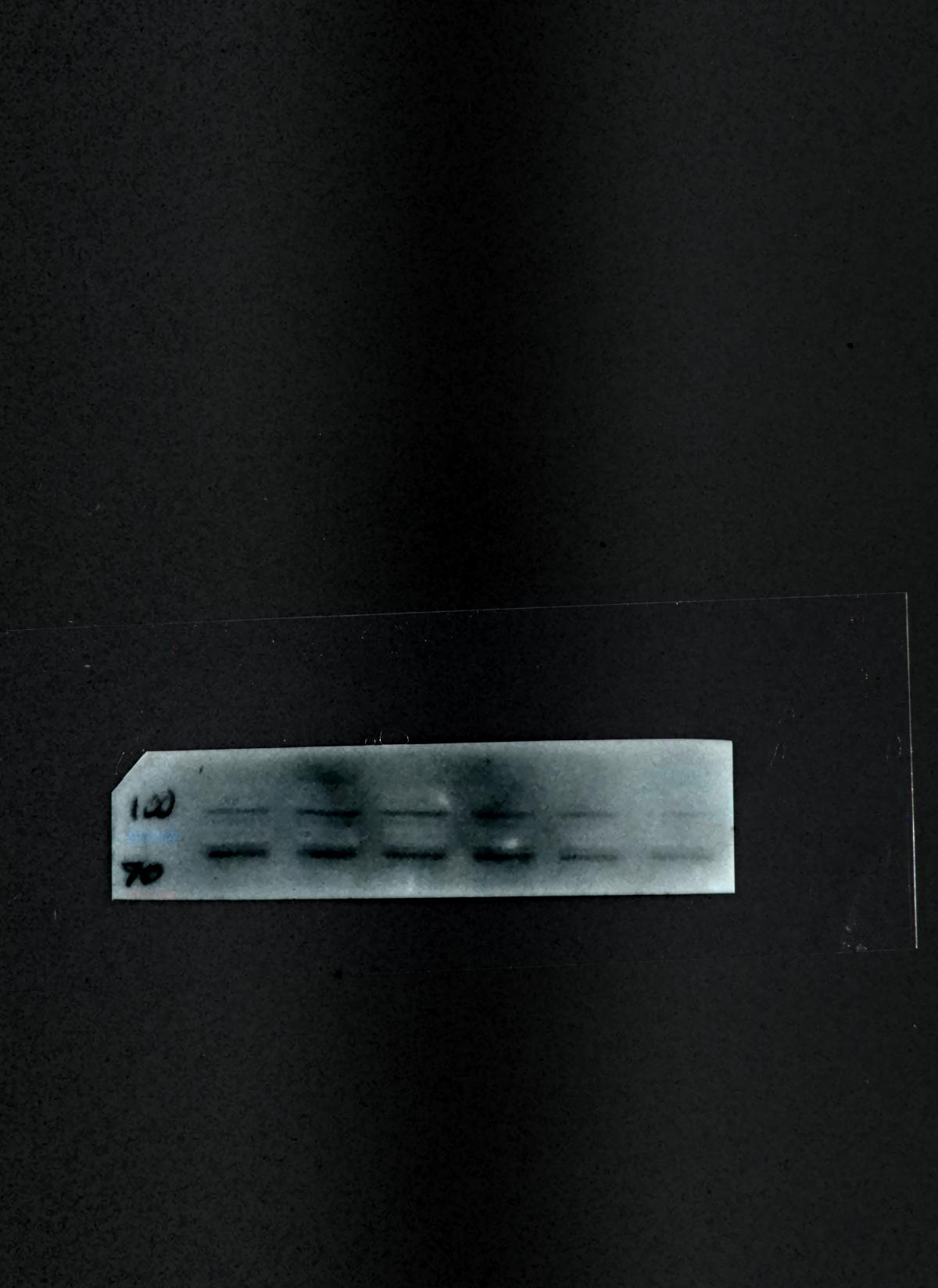 | 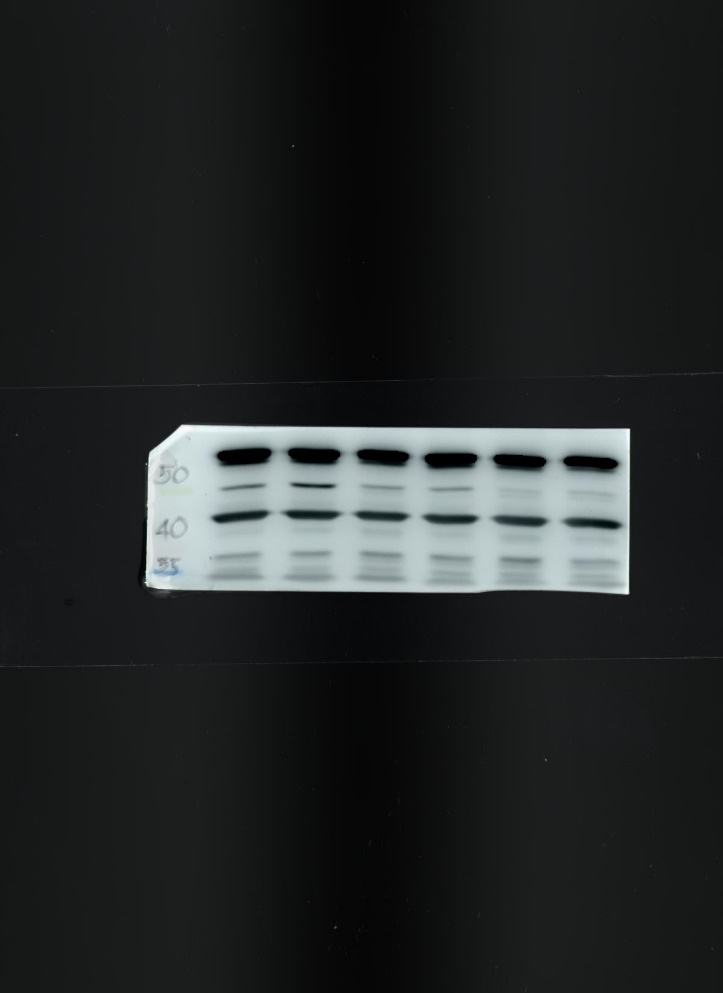 |
| KKU-213B | PI3K | 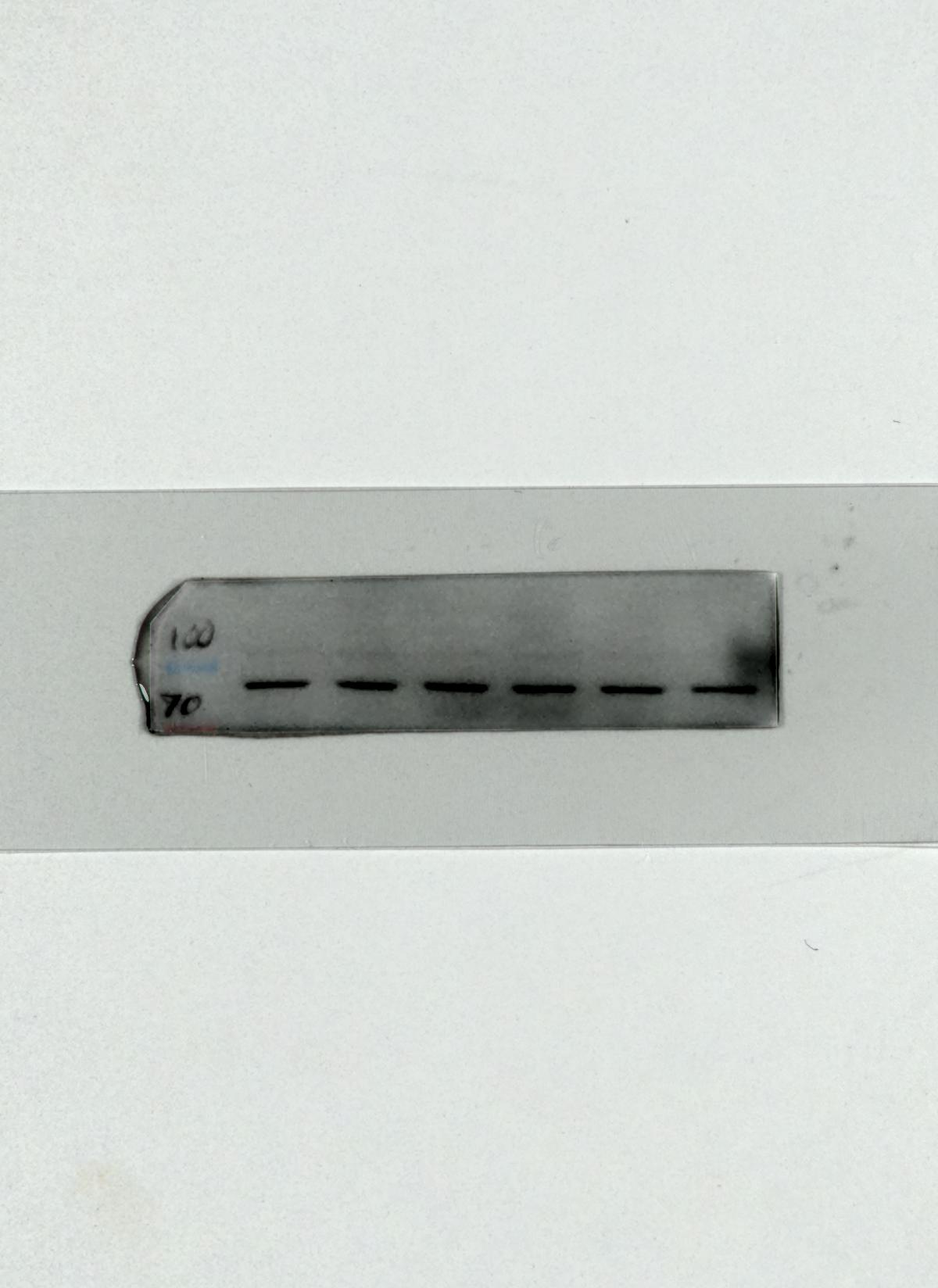 | 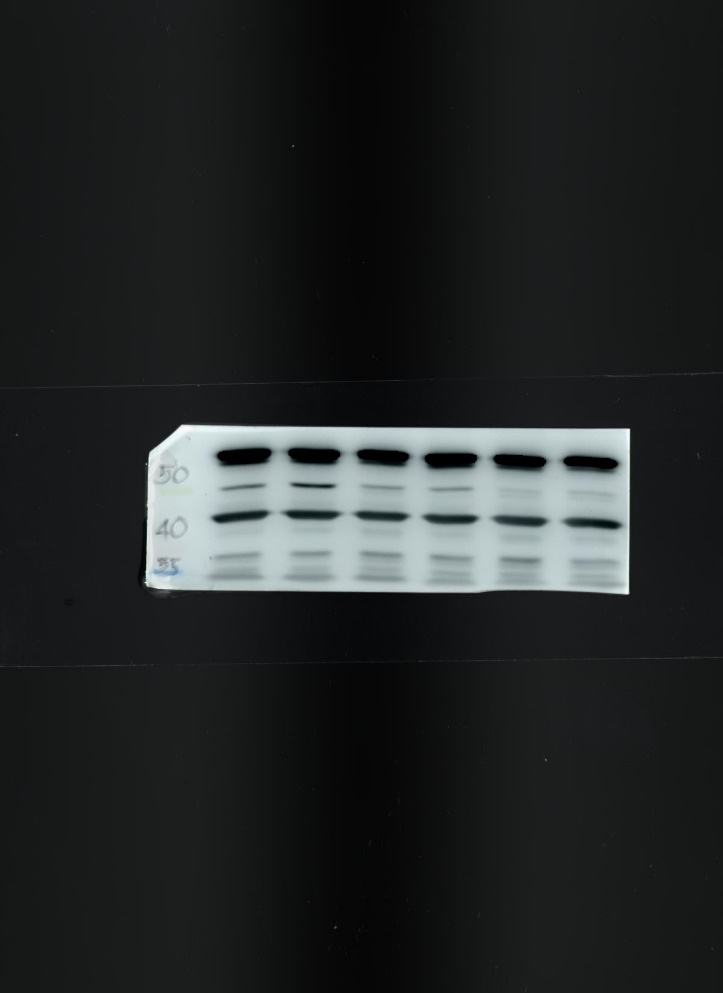 |
| KKU-213B | p-AKT | 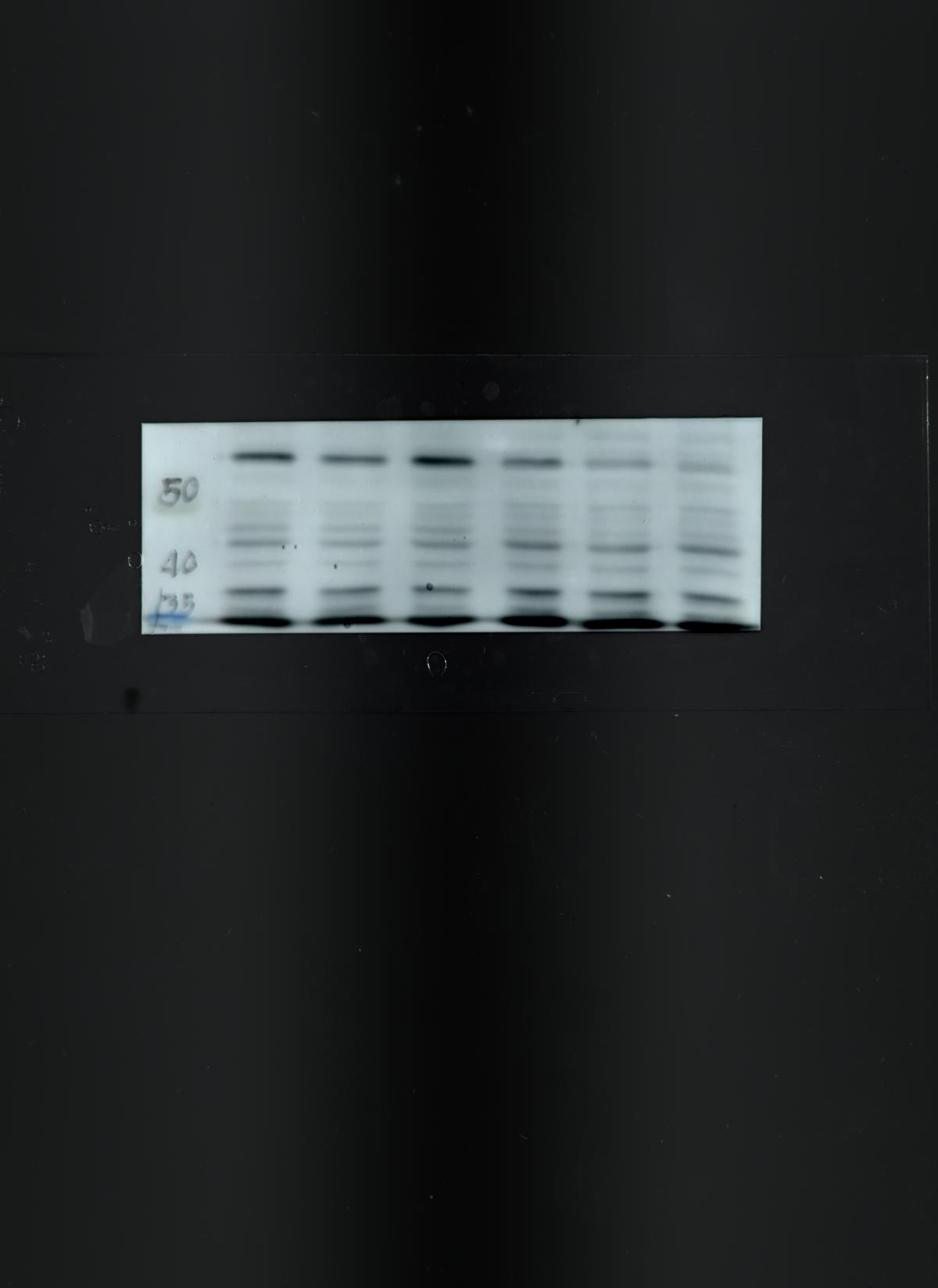 | 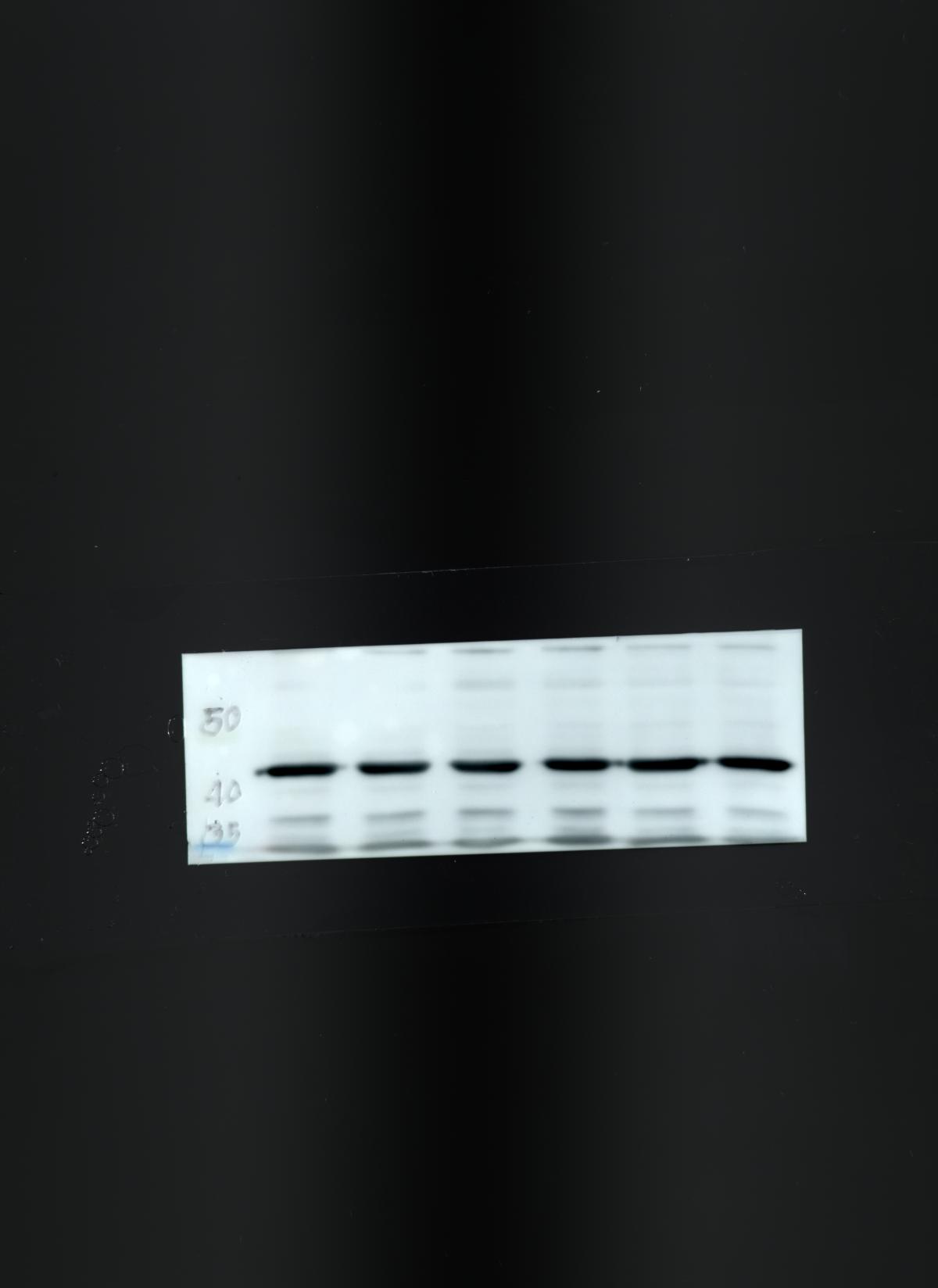 |
| KKU-213B | AKT | 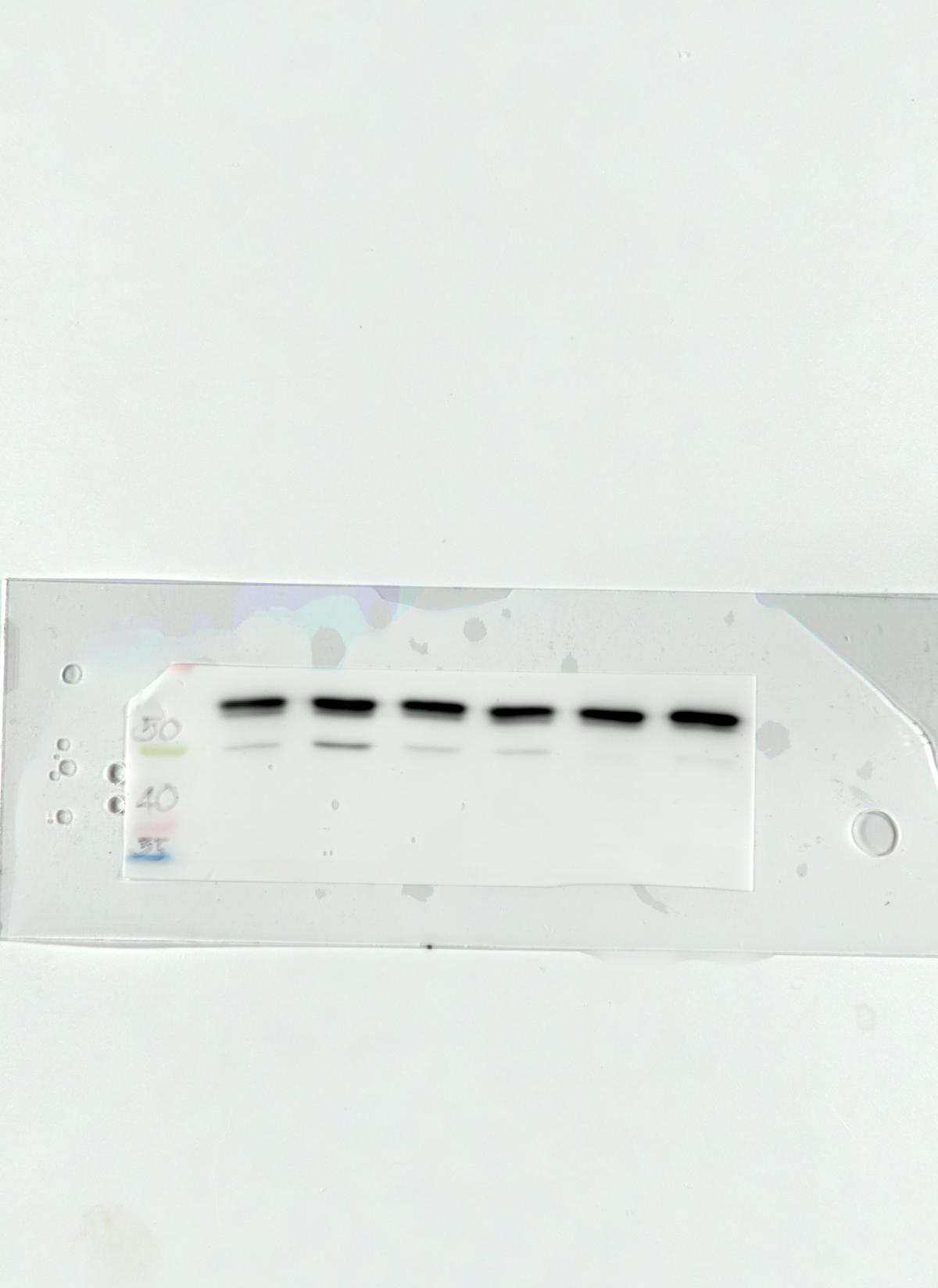 | 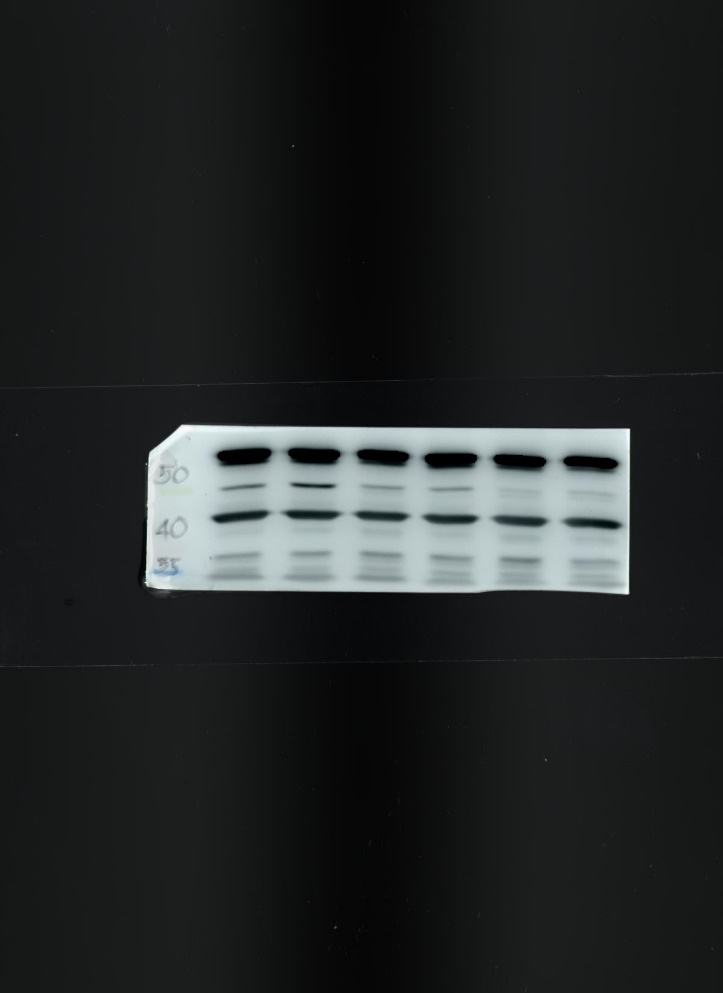 |
| KKU-213B | Bcl-2 | 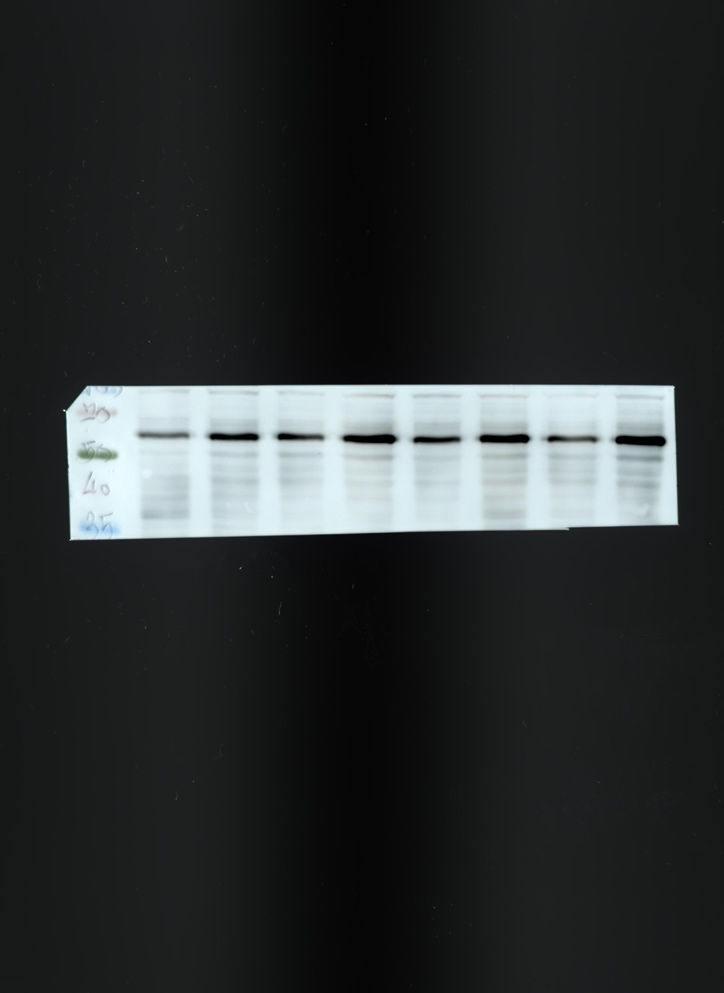 | 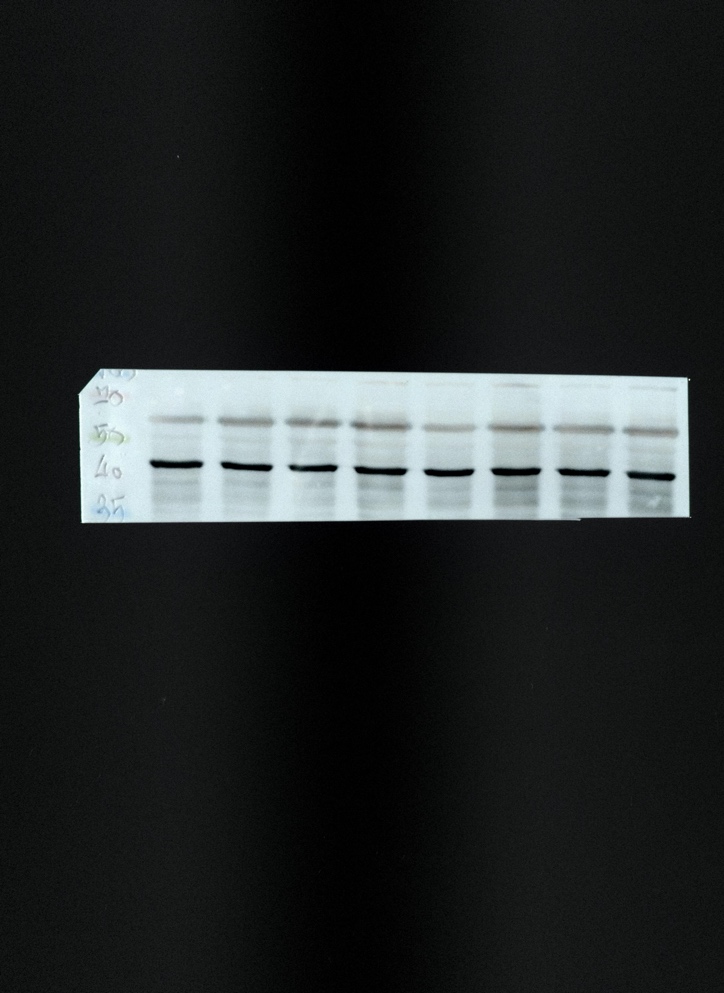 |
| KKU-100 | p-mTOR | 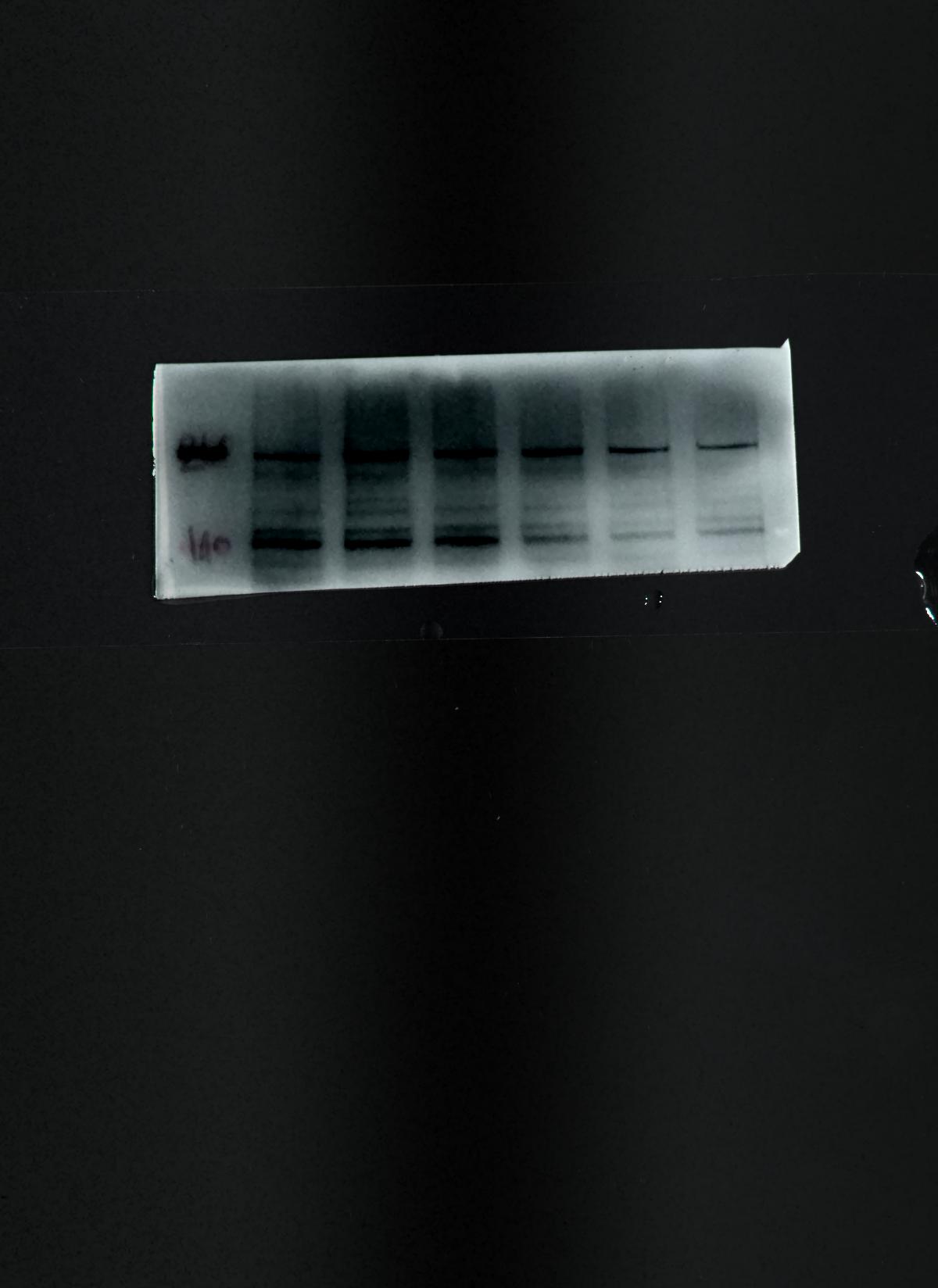 | 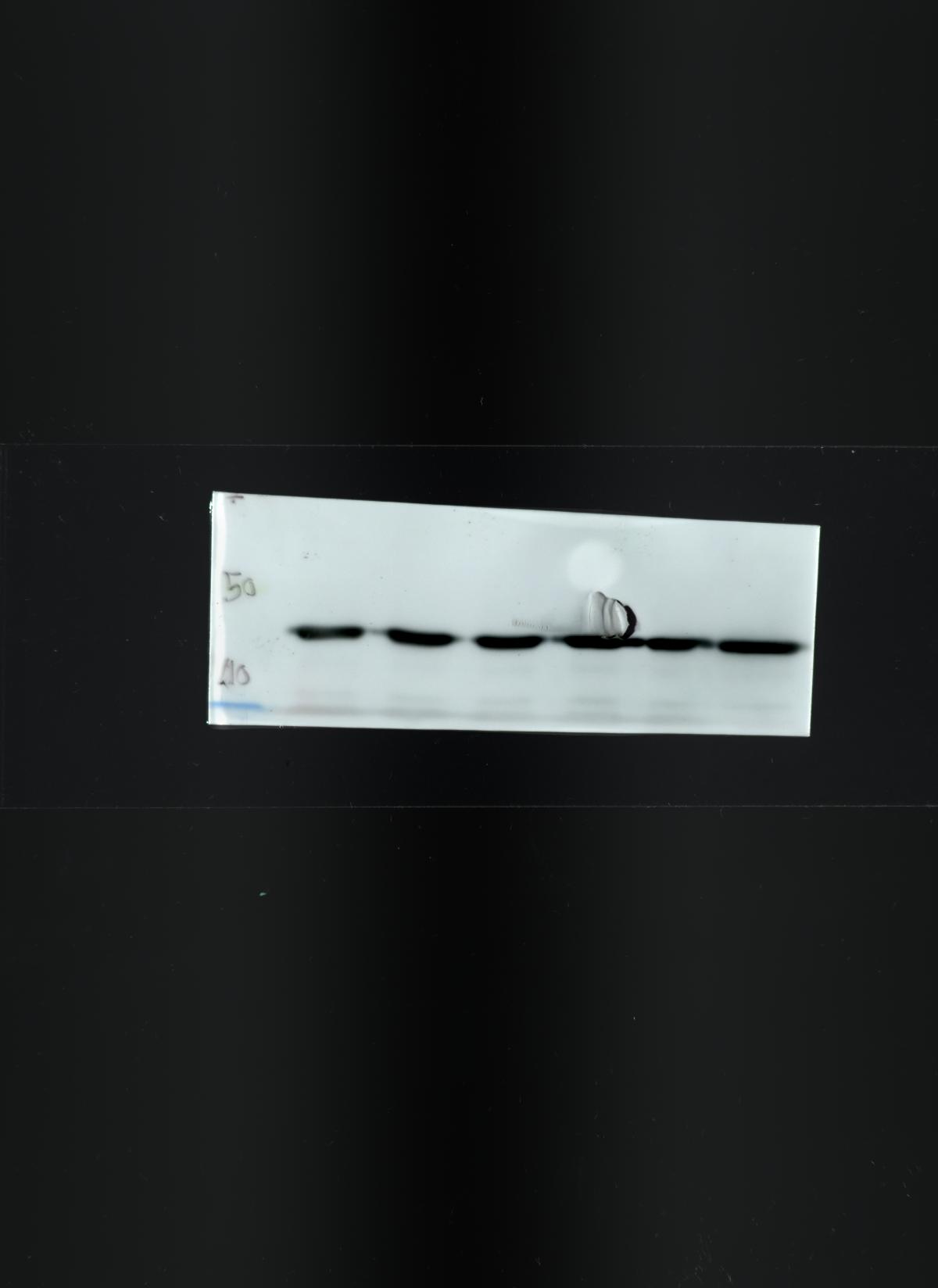 |
| KKU-100 | mTOR | 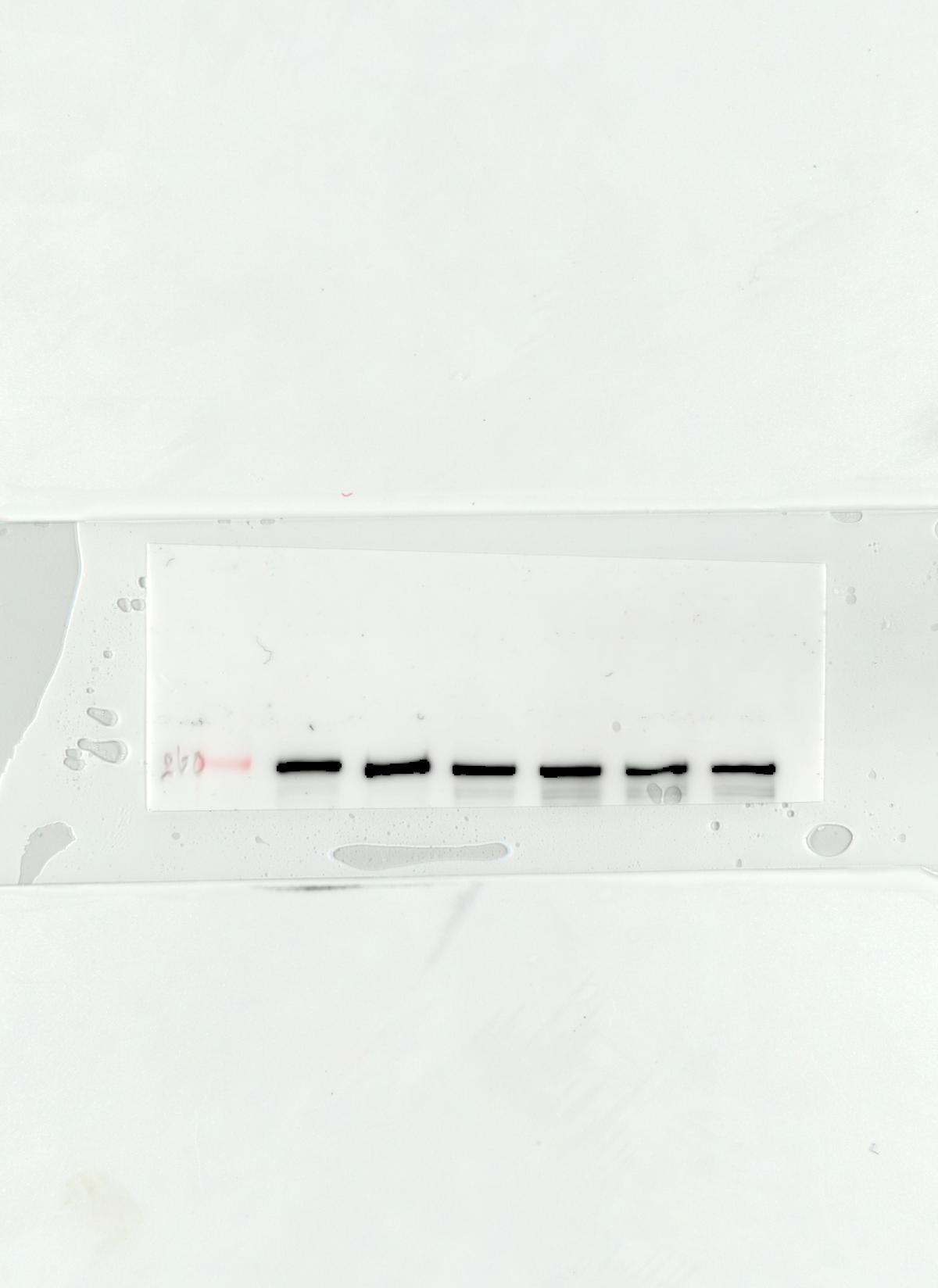 | 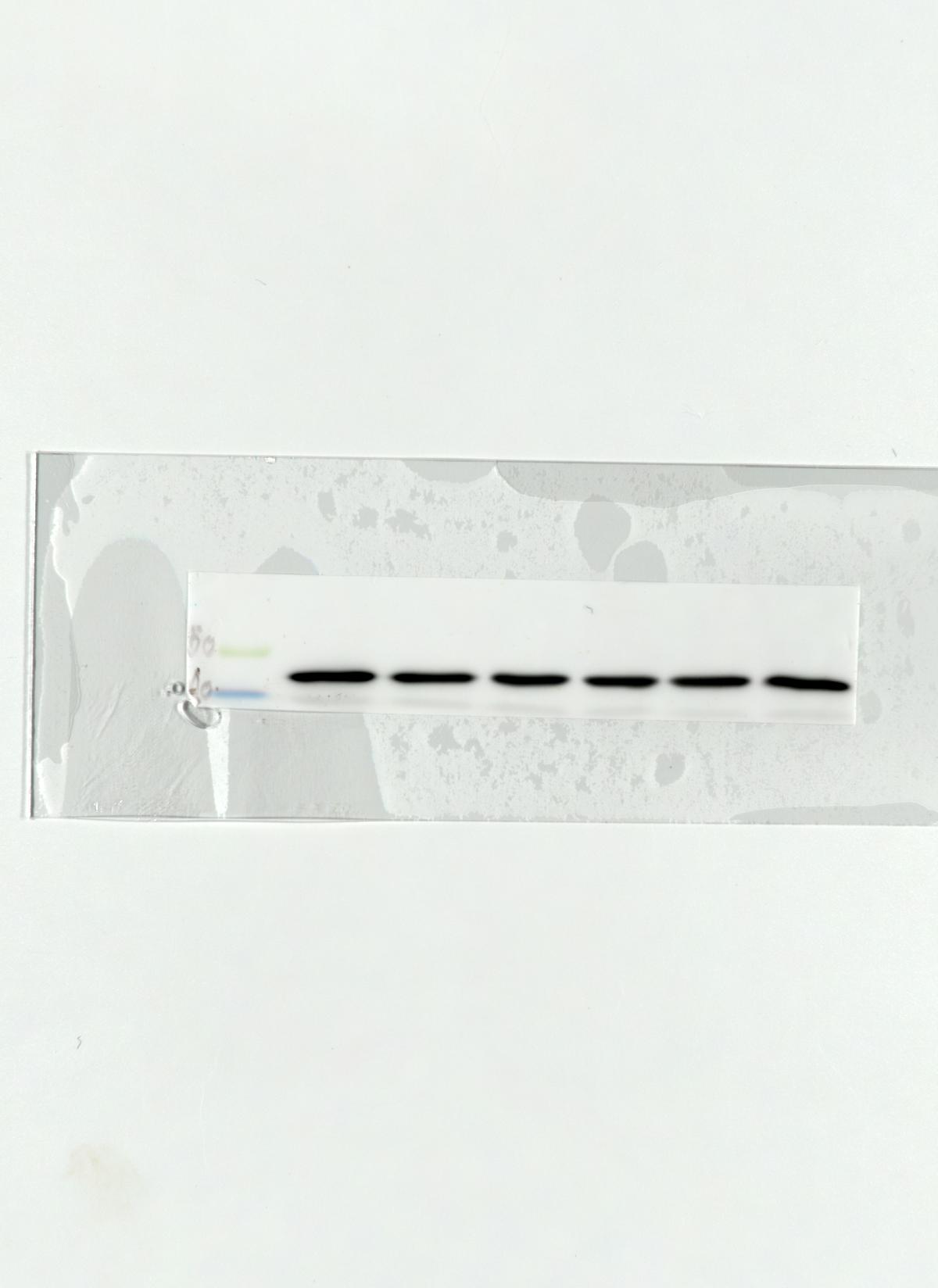 |
| KKU-100 | p-PI3K | 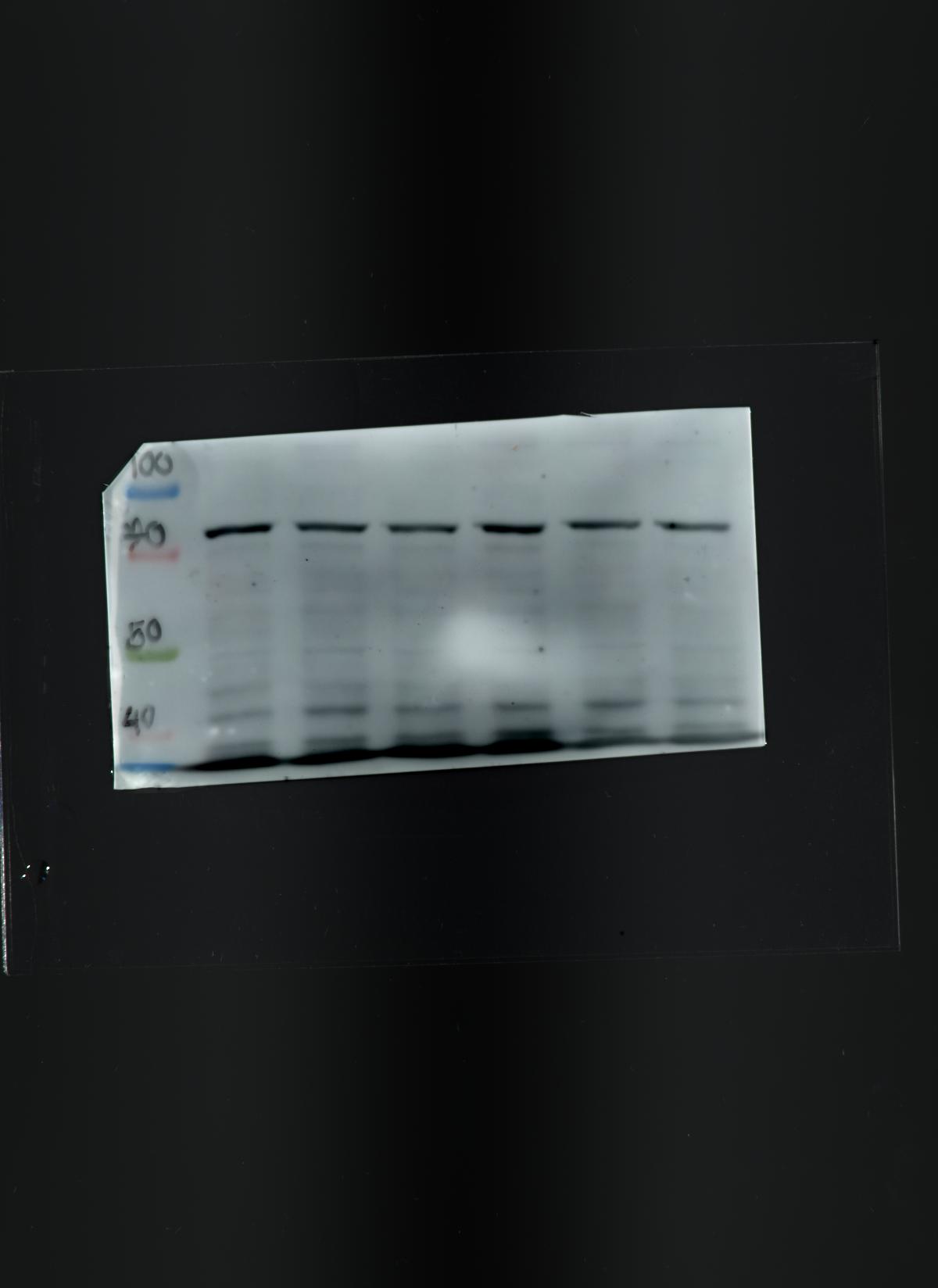 | 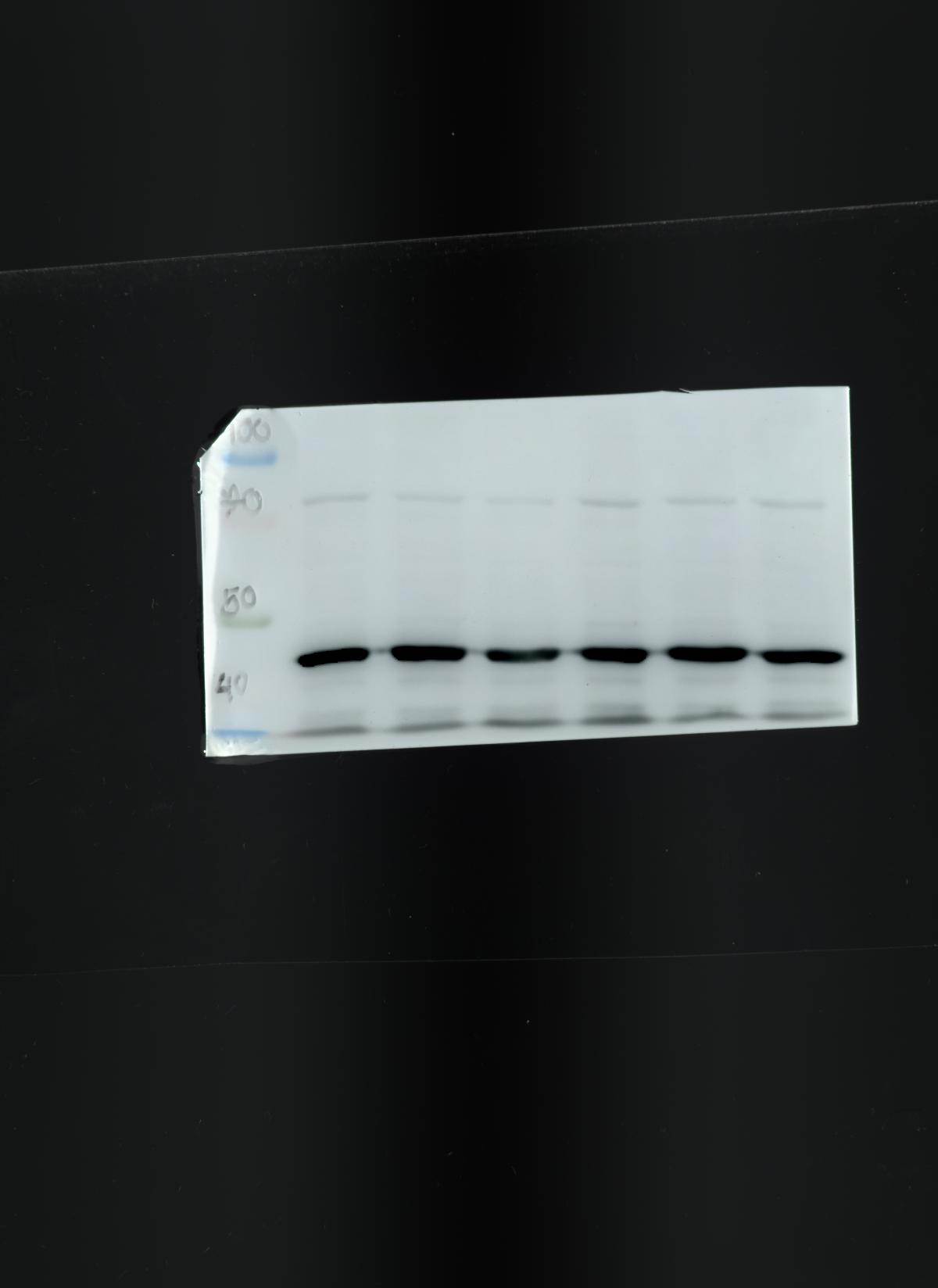 |
| KKU-100 | PI3K | 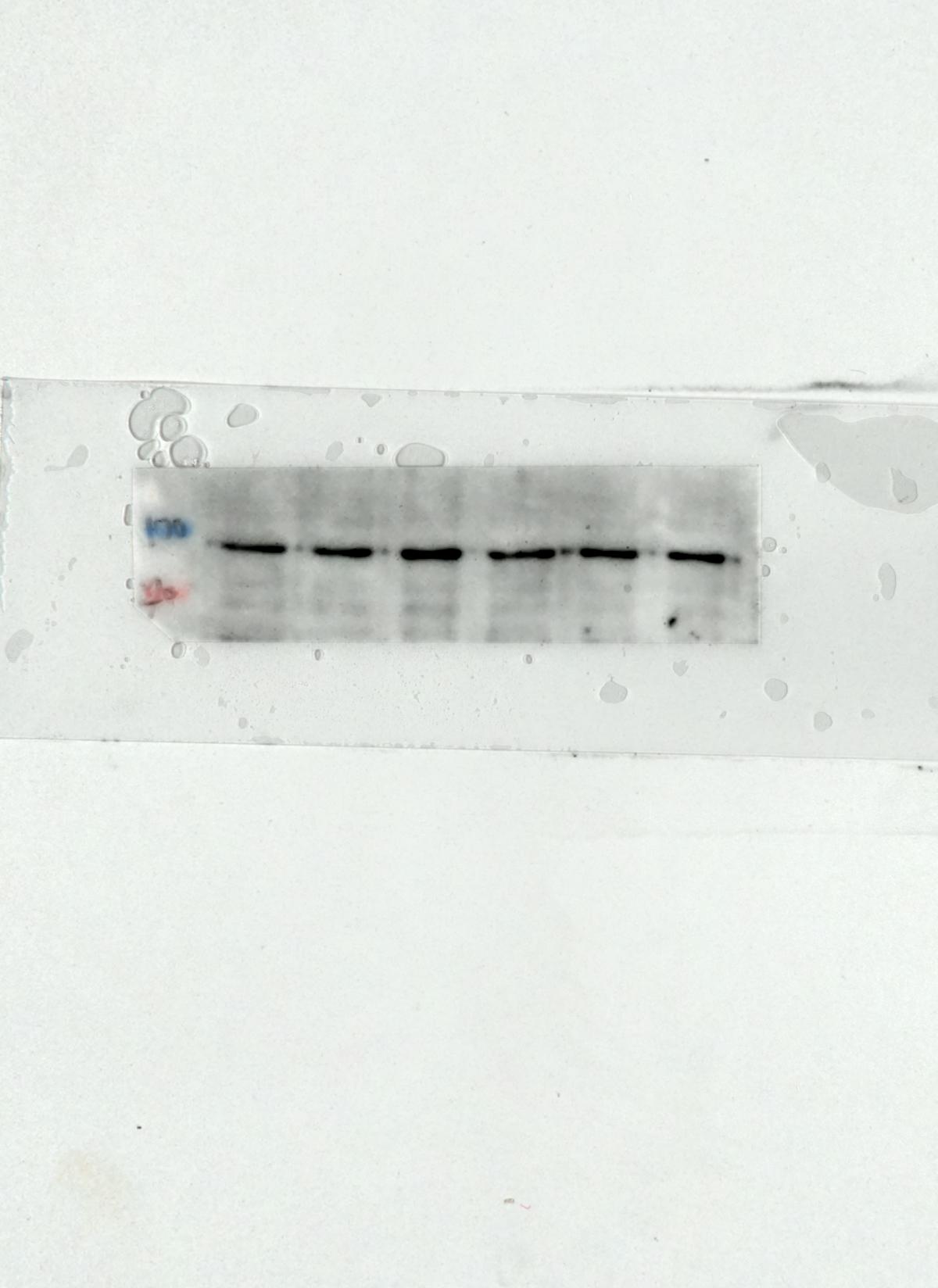 | 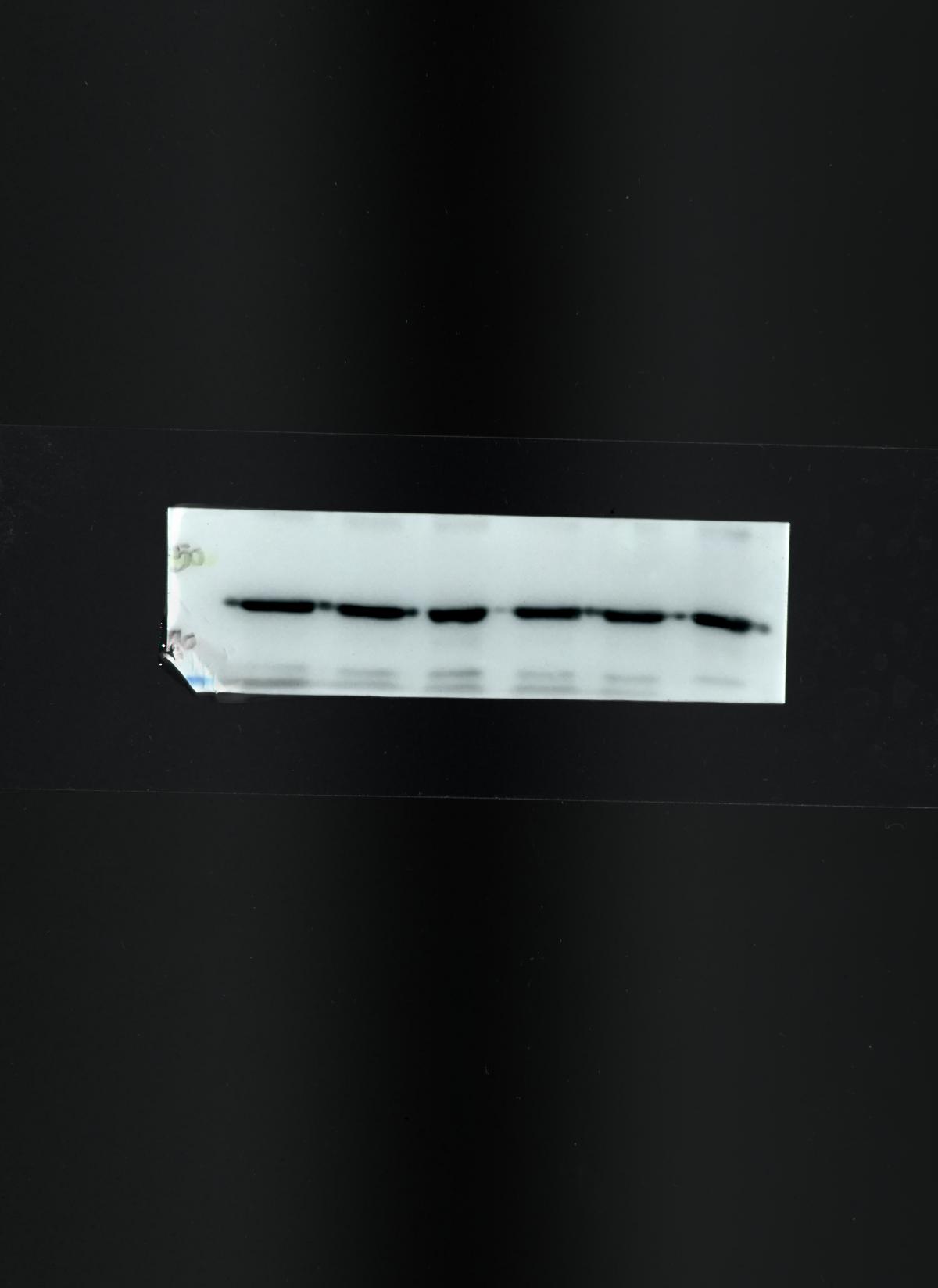 |
| KKU-100 | p-AKT | 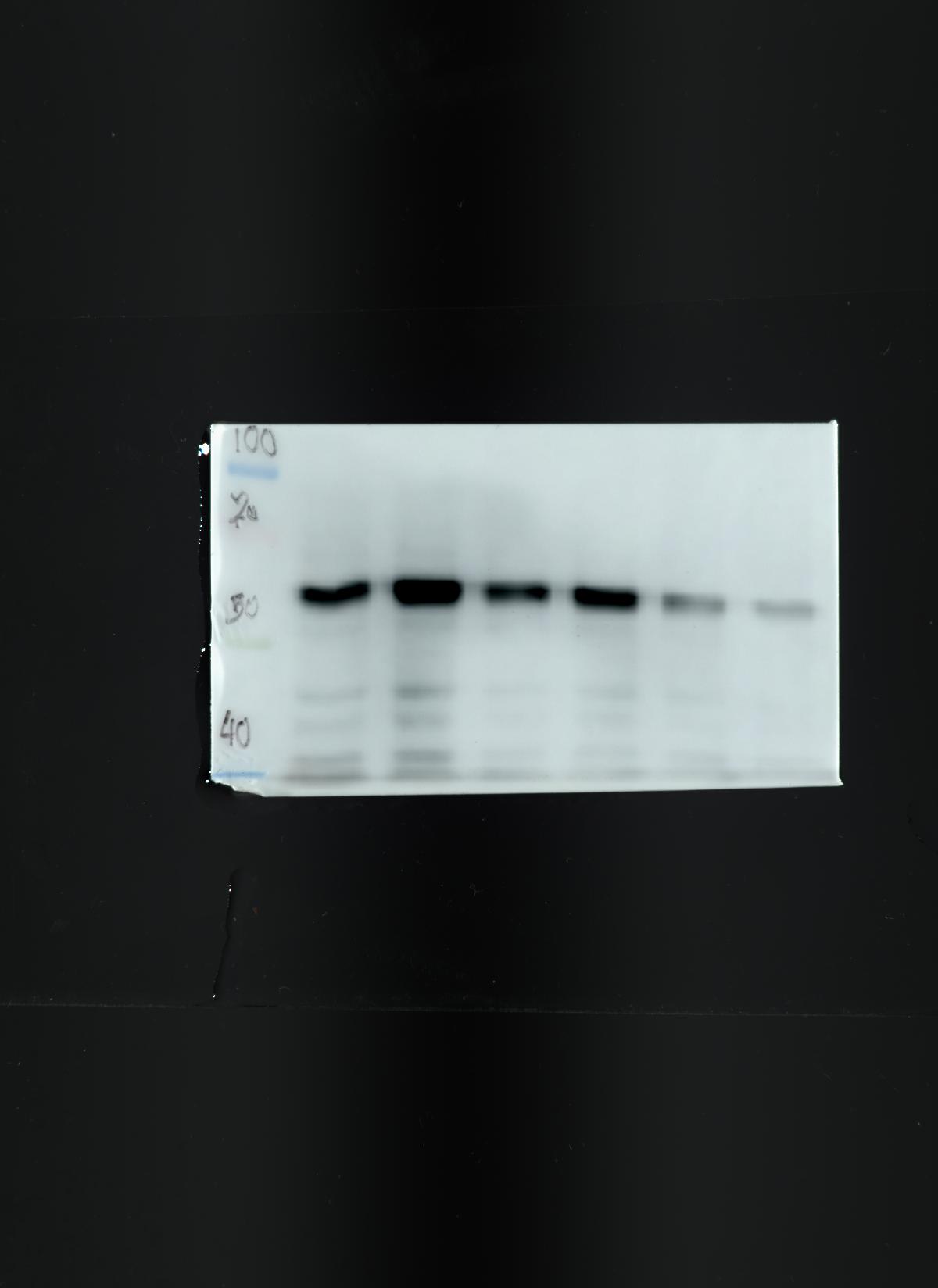 | 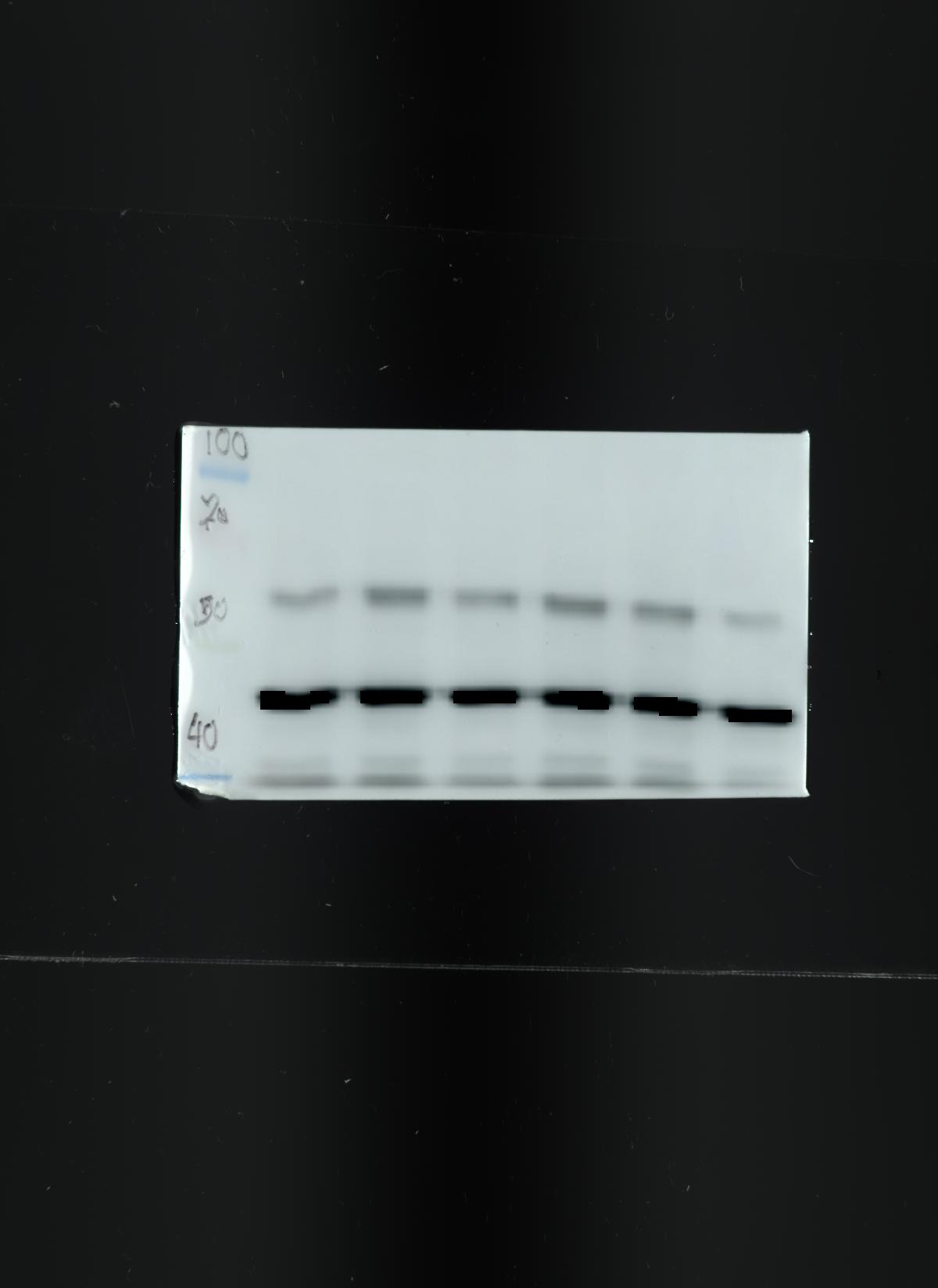 |
| KKU-100 | AKT | 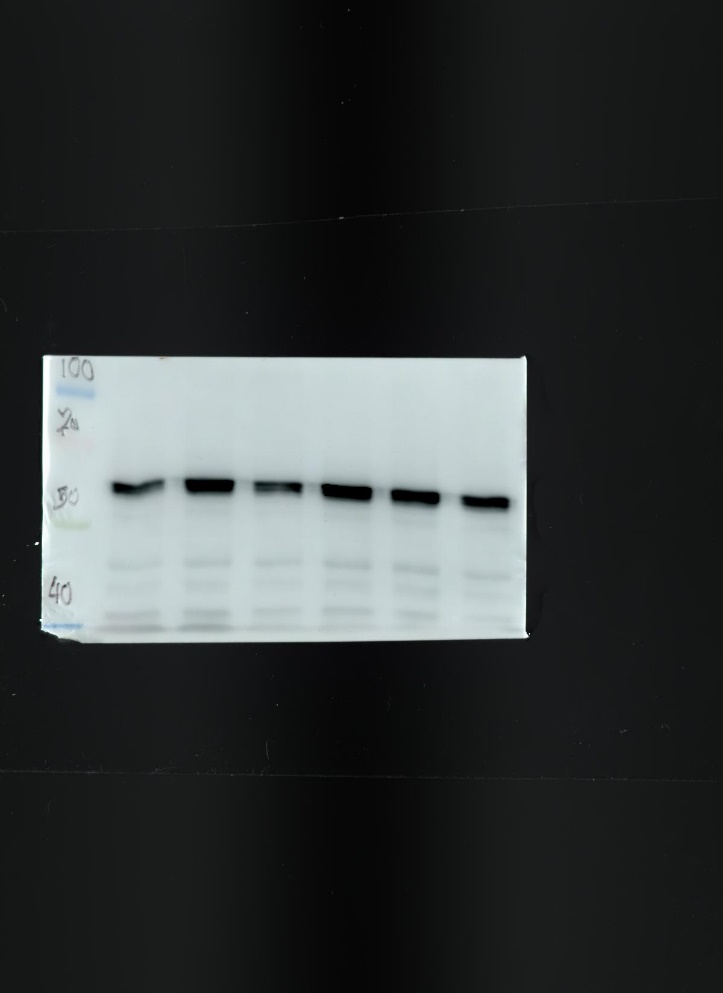 | 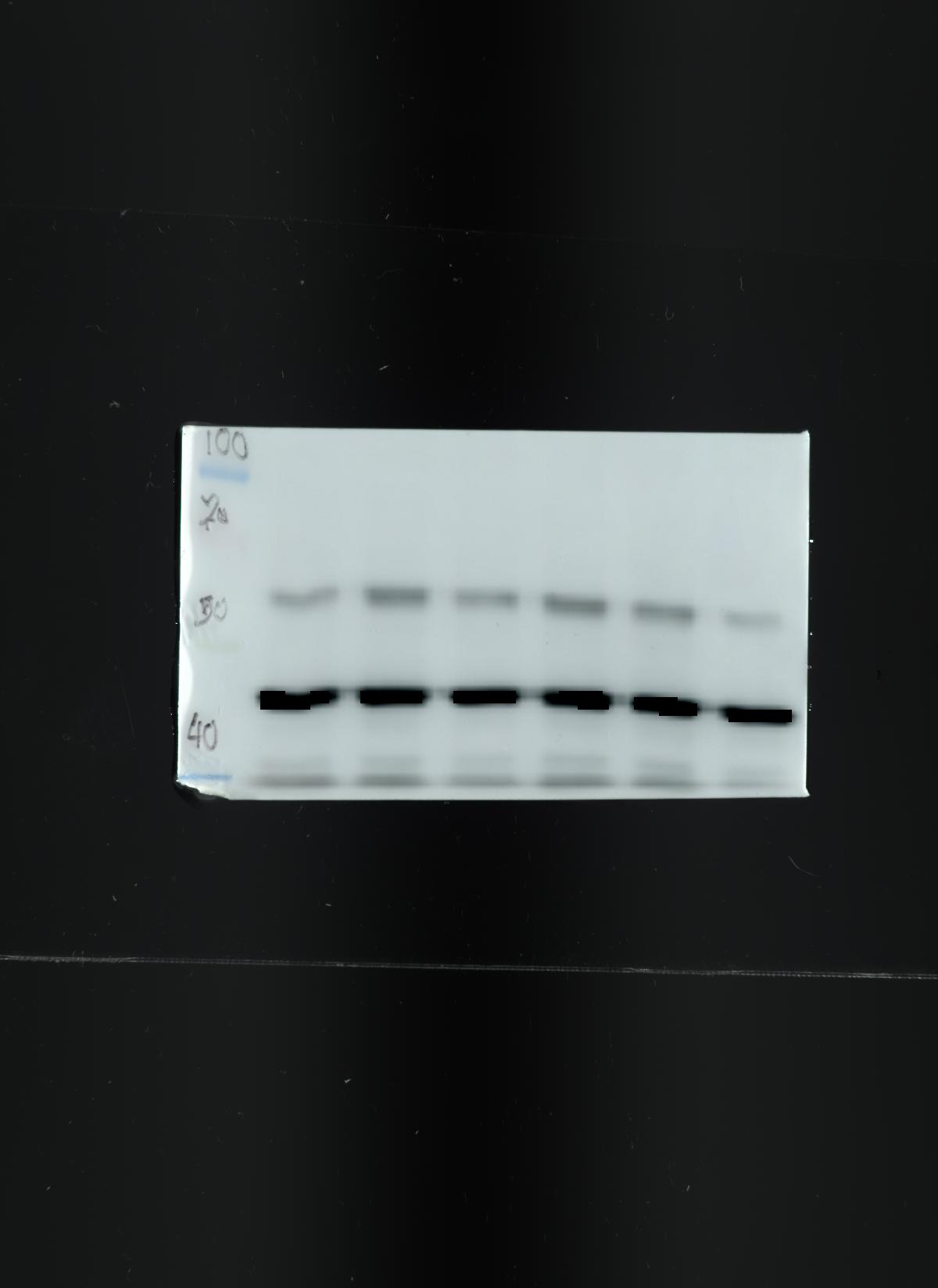 |
| KKU-100 | Bcl-2 | 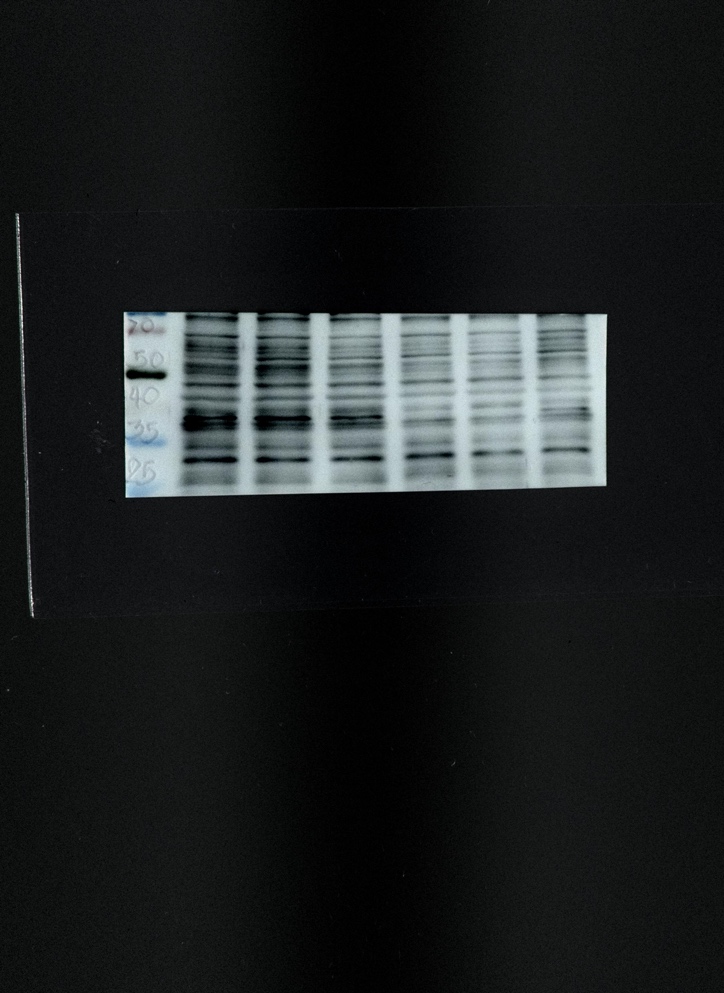 | 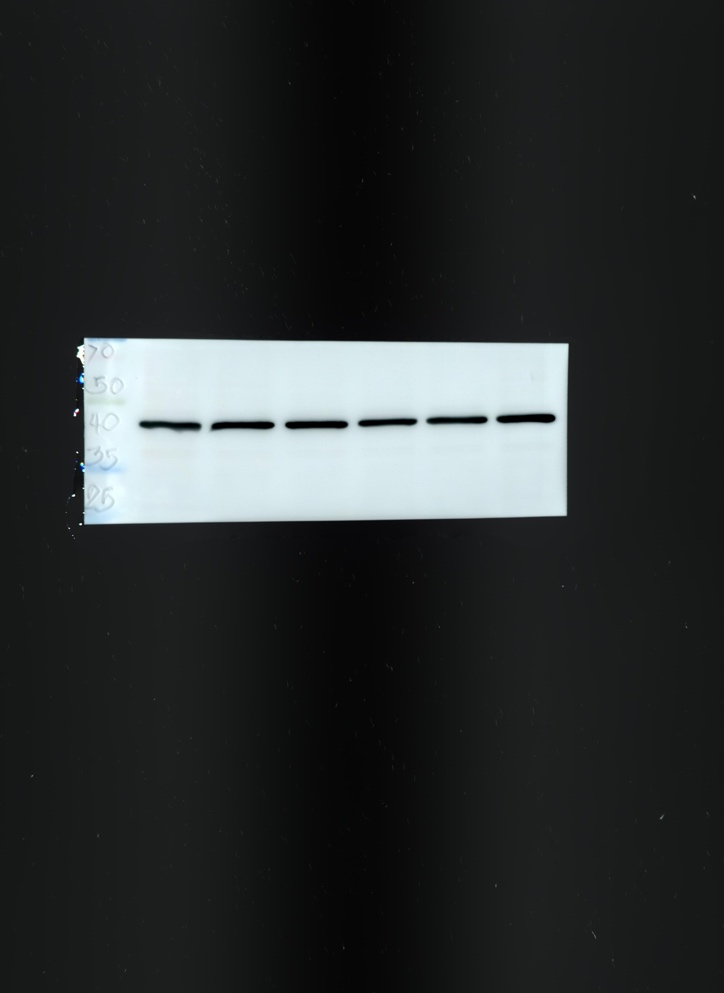 |

**Supplementary table 2** Original blotted membranes from KKU-213B and KKU-100 cell lines following treatment with varying concentrations of chloroquine (CQ: 10 µM) and/or CBD (10 µM) for 48 h. A control group, treated with 0.3% DMSO, served as the vehicle control for investigating autophagy-associated markers (LC3B and p62). Additionally, a subset of cells was pre-treated with CQ at a concentration of 10 µM, administered 8 h prior to CBD exposure. In the western blot analysis, lane 1 represents the molecular weight marker, while lanes 6 through 9 depict samples from the CCA cell lines treated with 0.3% DMSO, CQ at 10 µM, CBD at 10 µM, and CQ plus CBD for 48 h, respectively.

| Cell lines | Protein target | Original membranes of target protein | Beta-actin |
| --- | --- | --- | --- |
| KKU-213B | LC3B | 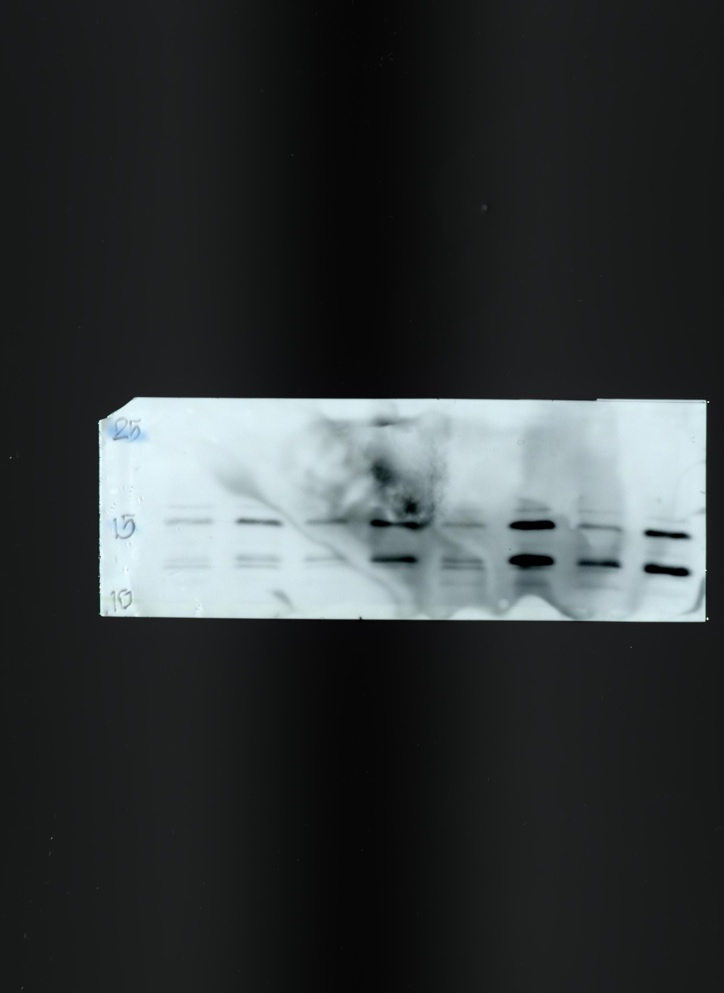 | 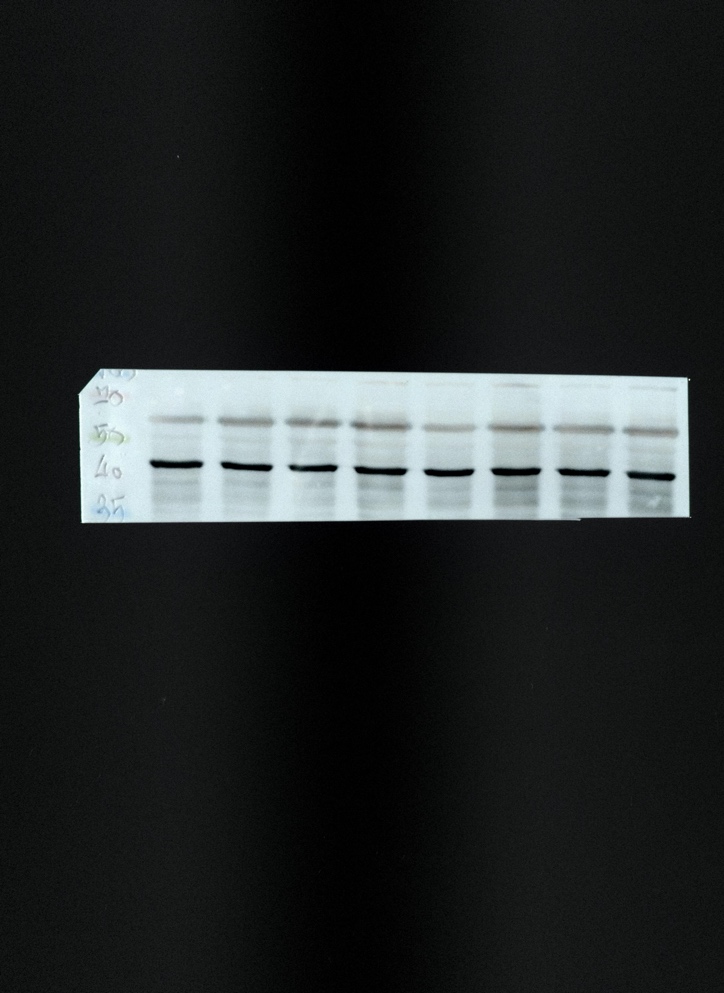 |
| KKU-213B | Bcl-2 | 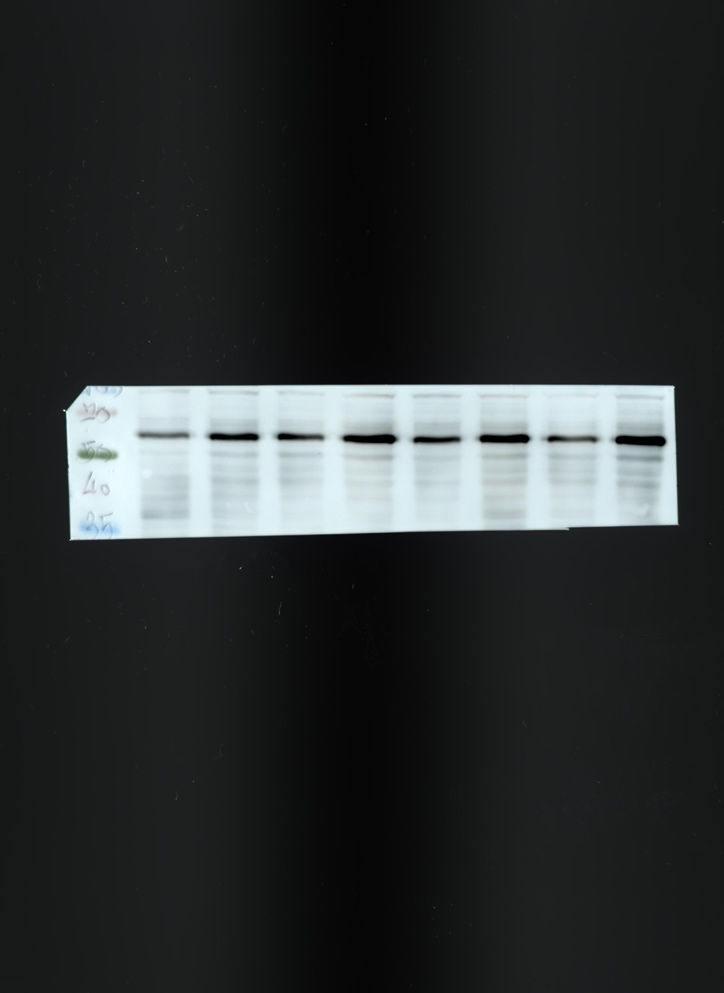 | 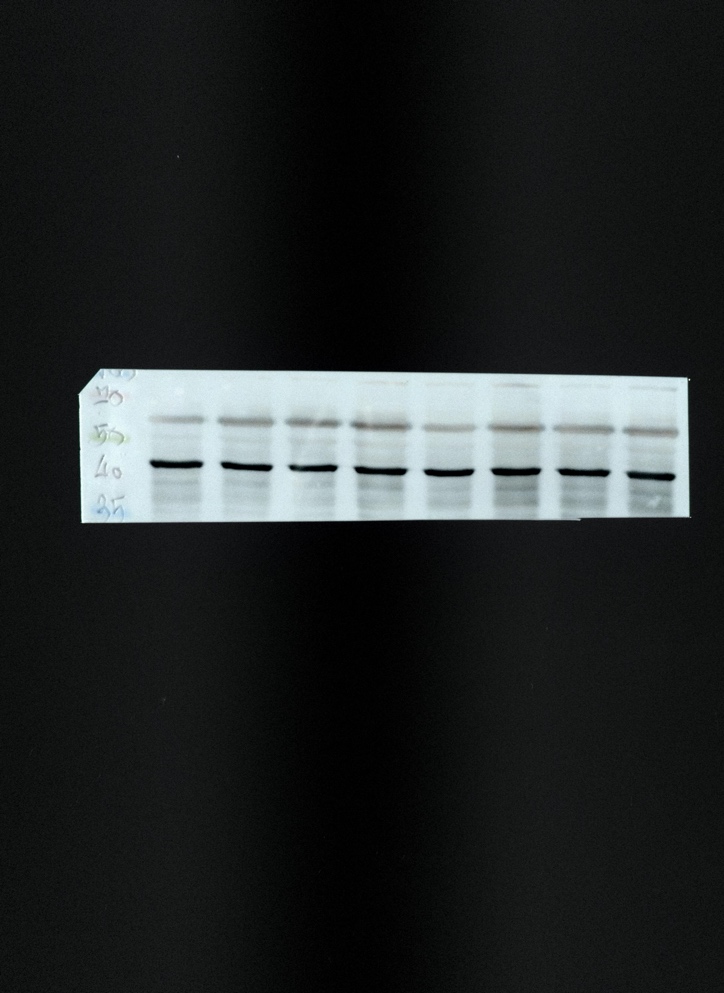 |

| KKU-100 | LC3B | 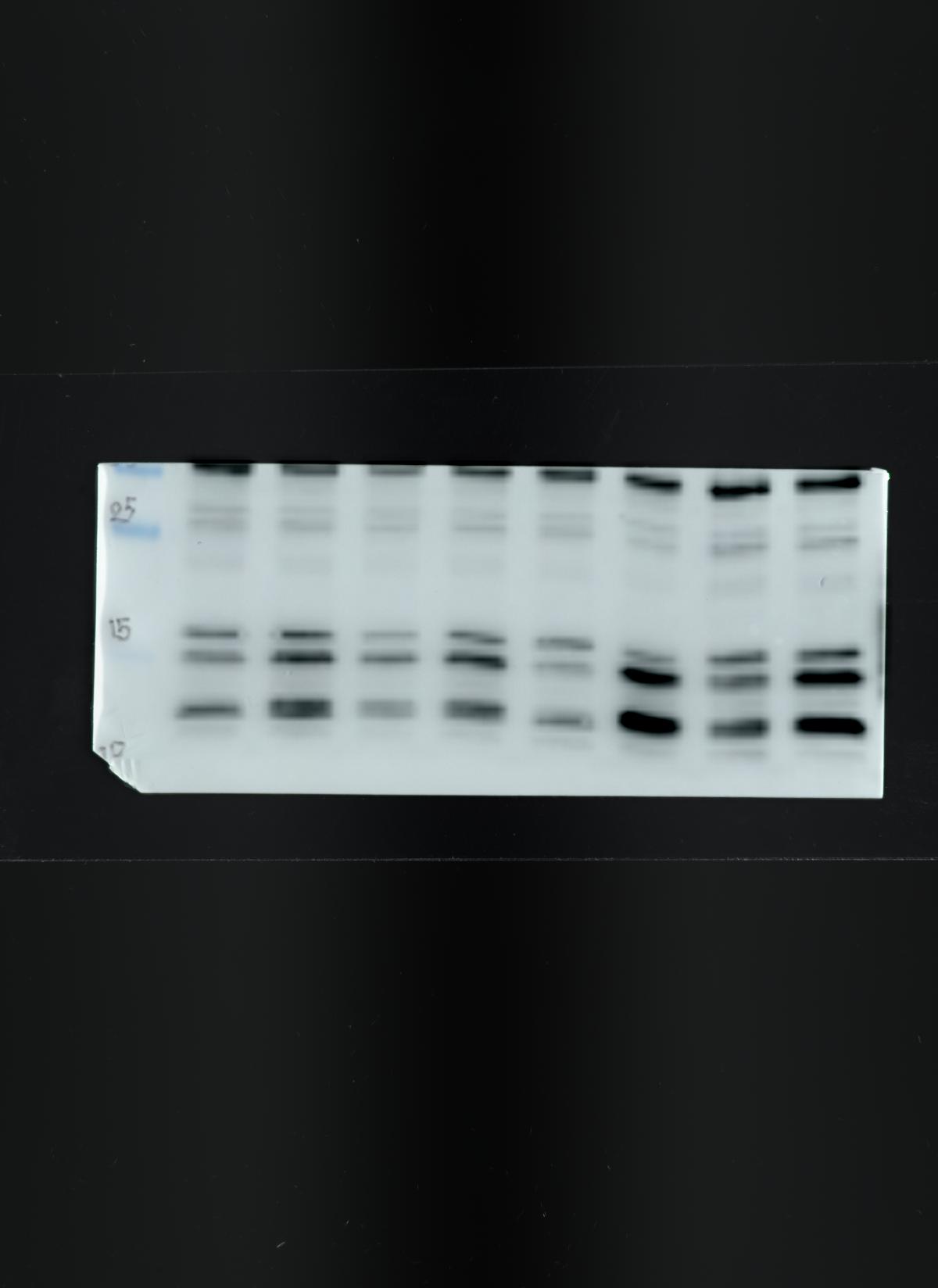 | 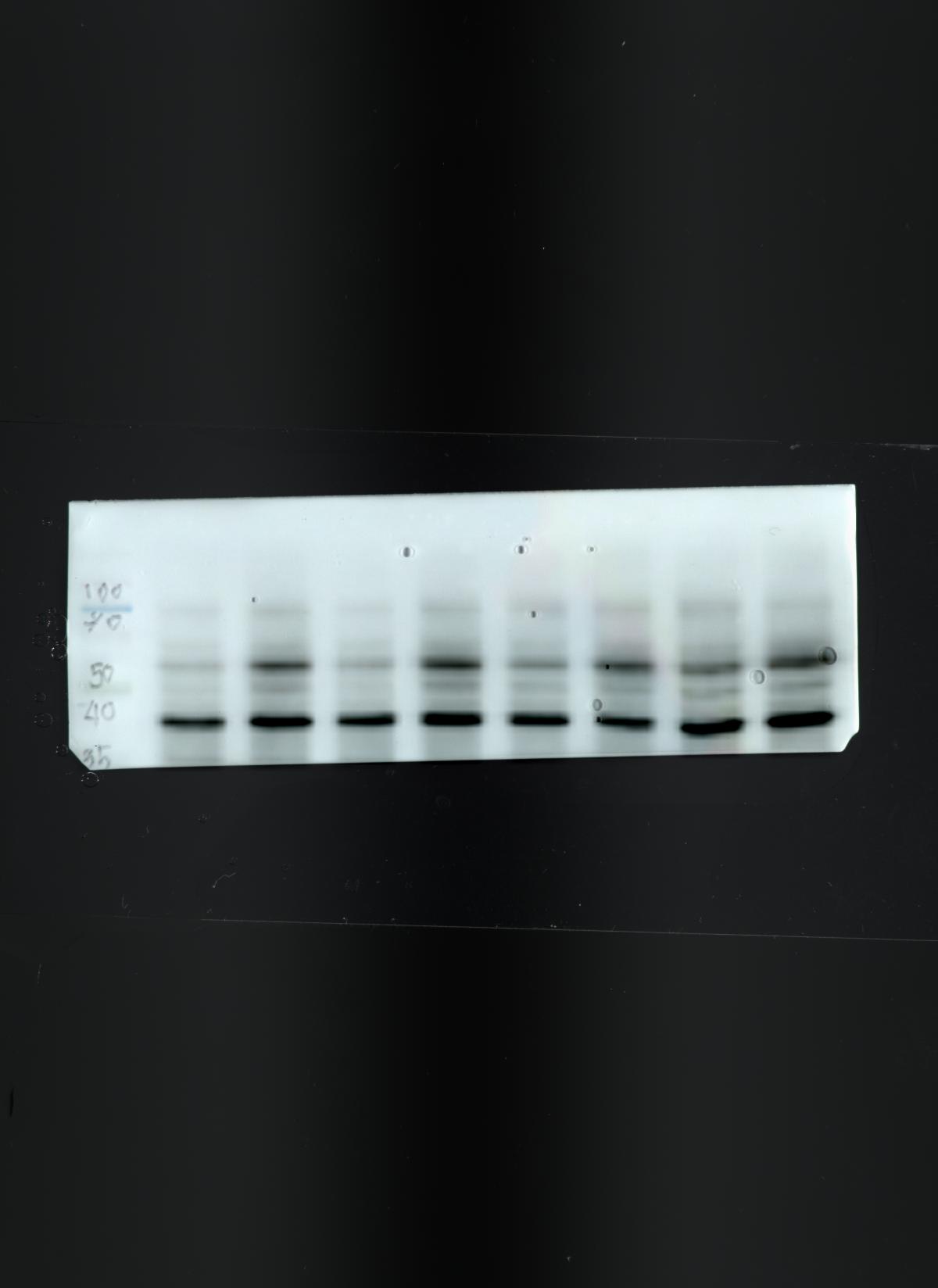 |
| --- | --- | --- | --- |
| KKU-100 | p62 | 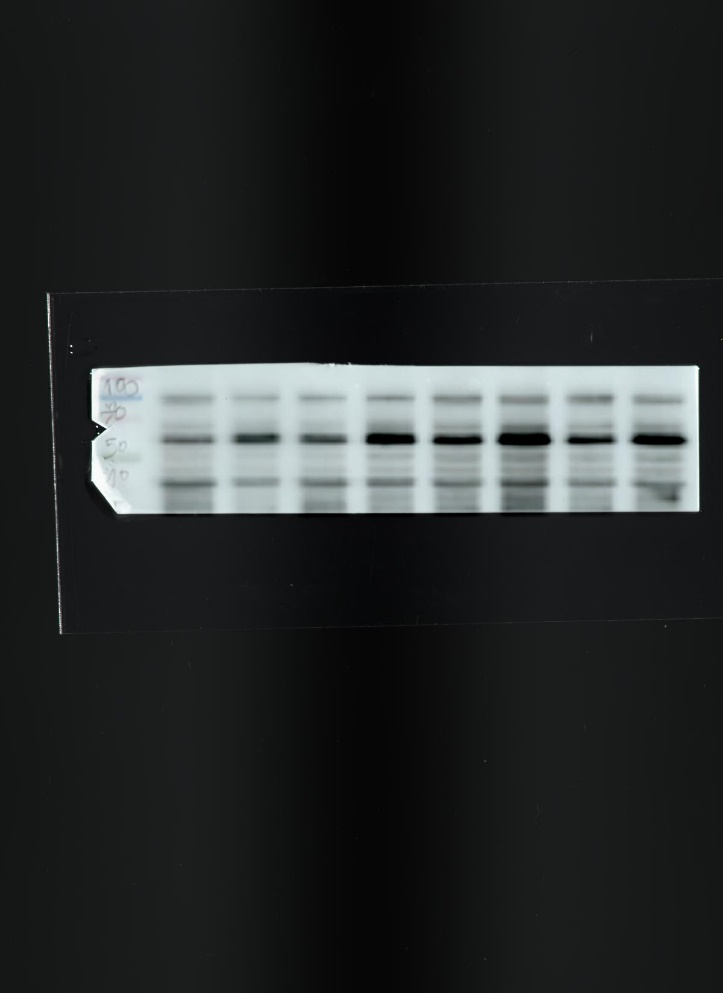 | 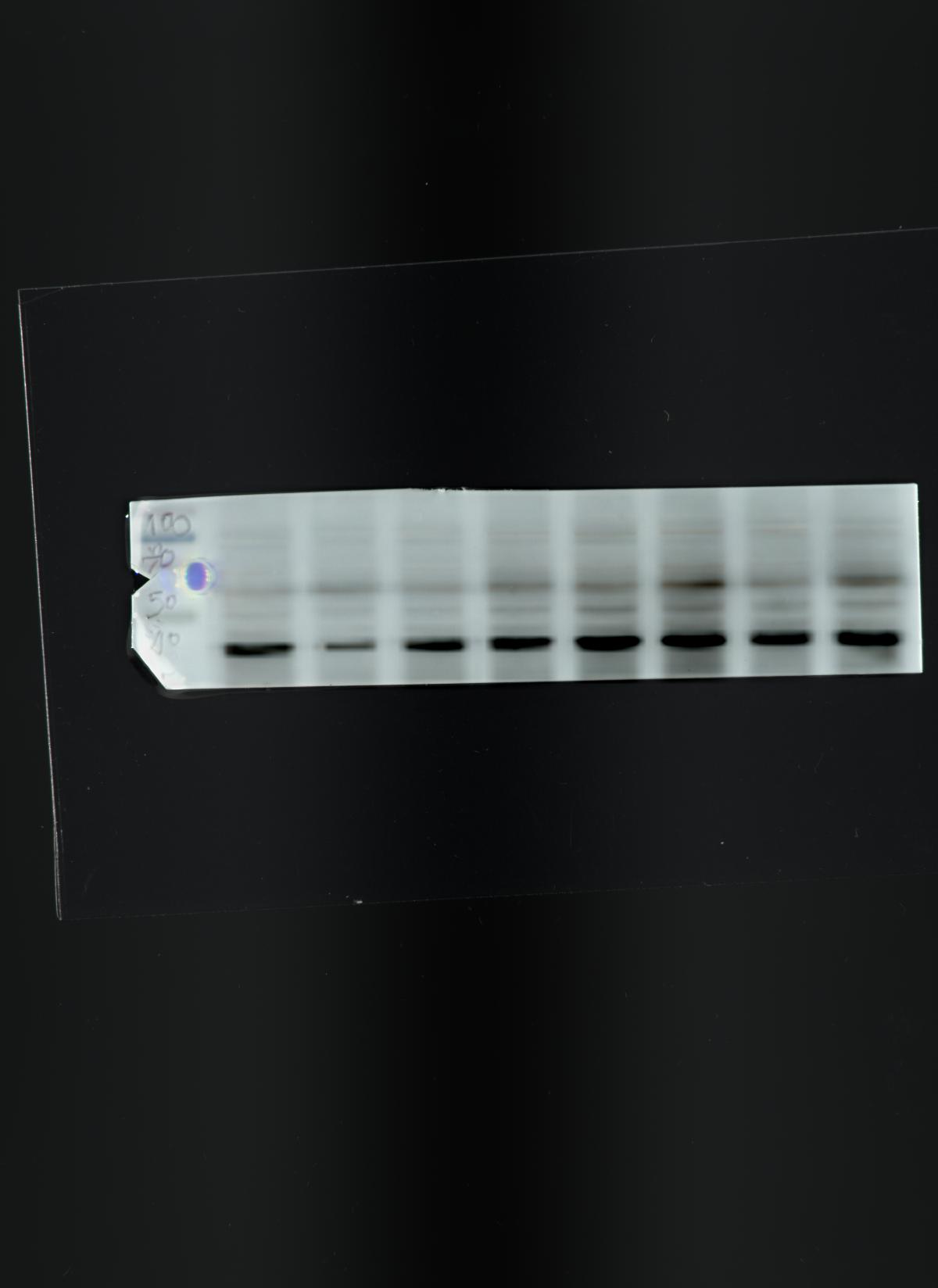 |

**Supplementary table 3** Original blotted membranes from KKU-213B and KKU-100 cell lines following treatment with varying concentrations of CBD (5 µM and 10 µM) for 24 h. A control group, treated with 0.3% DMSO, served as the vehicle control for investigating cellular senescence-associated markers (p21 and p53). In the western blot analysis, lane 1 represents the molecular weight marker, while lanes 2 through 9 depict samples from the CCA cell lines treated with 0.3% DMSO, CQ at 10 µM, CBD at 10 µM, and CQ plus CBD for 48 h, respectively.

| Cell lines | Protein target | Original membranes of target protein | Beta-actin |
| --- | --- | --- | --- |
| KKU-213B | p53 | 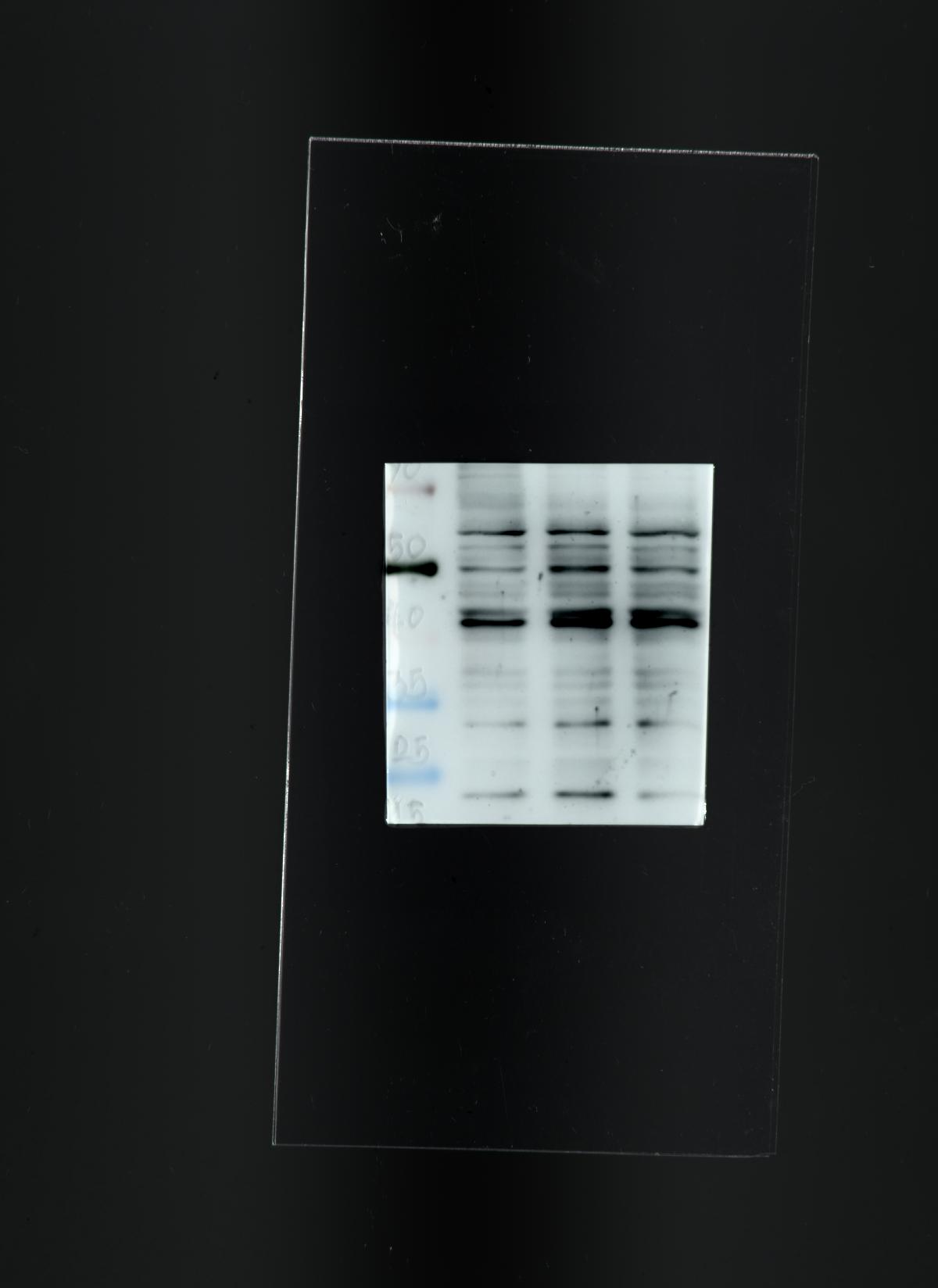 | 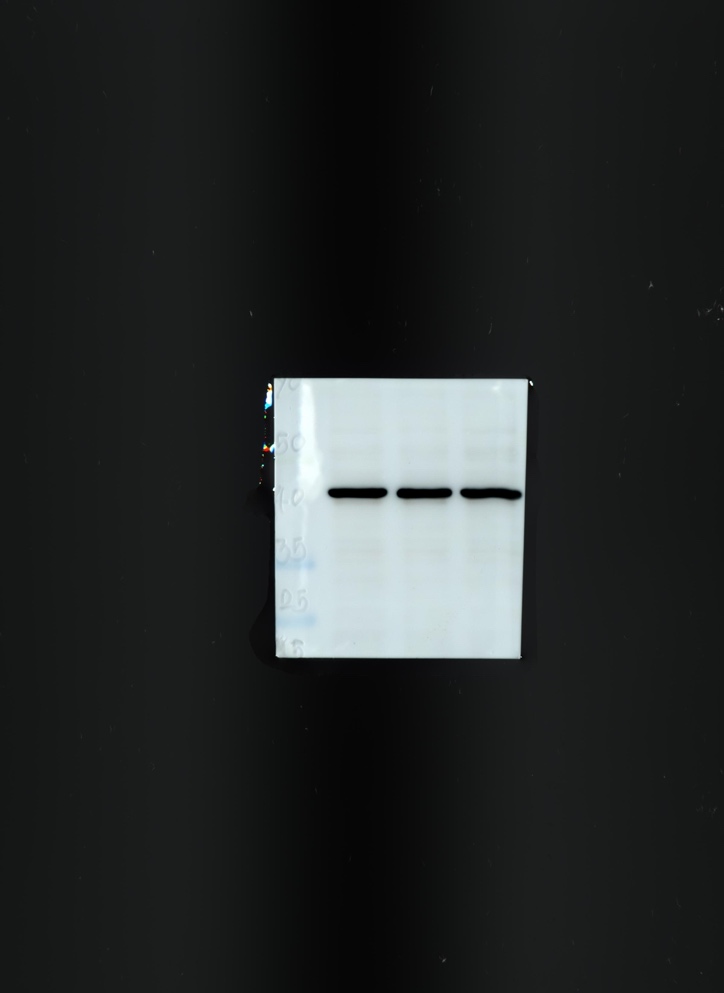 |
| KKU-213B | p21 | 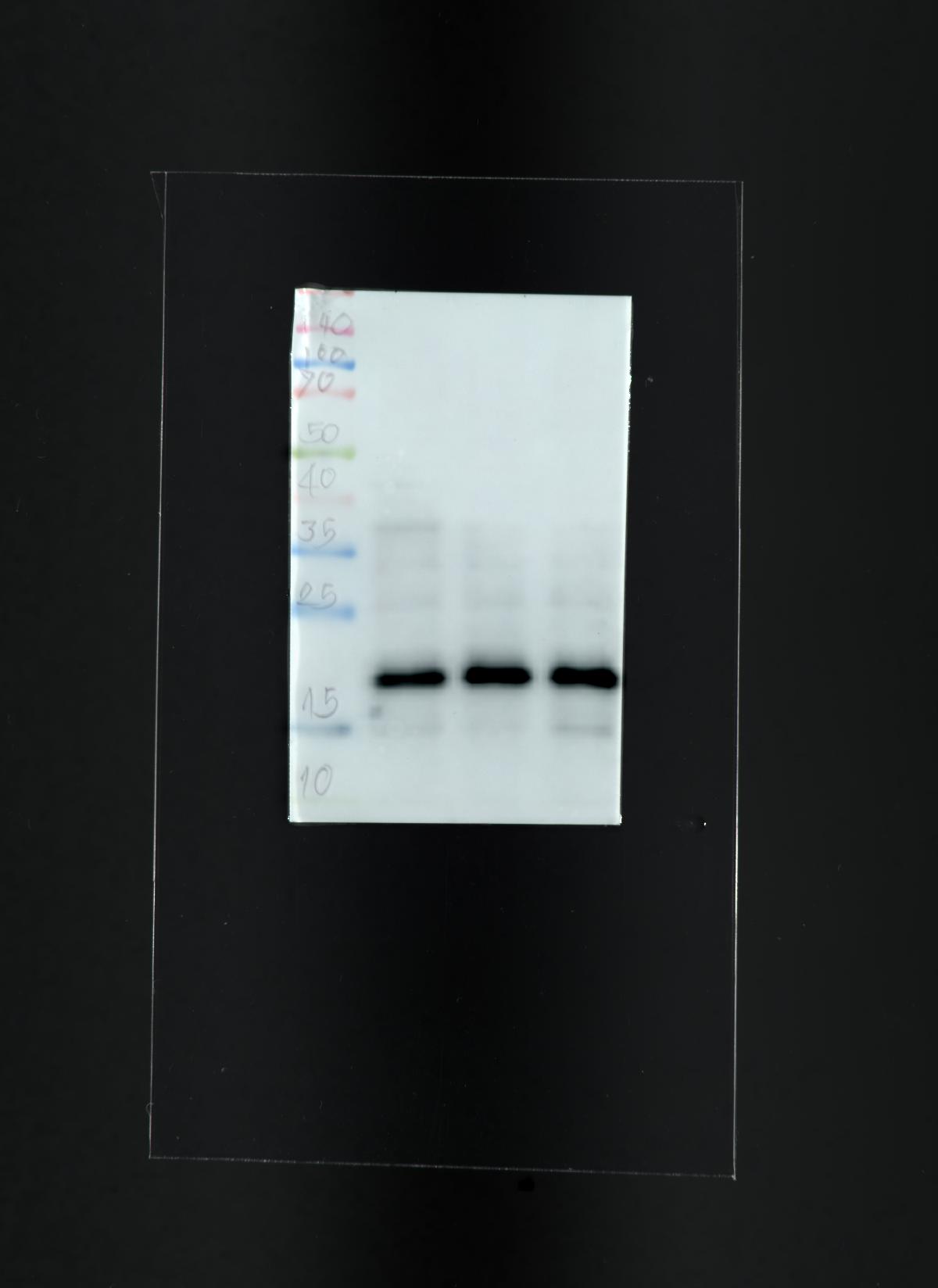 | 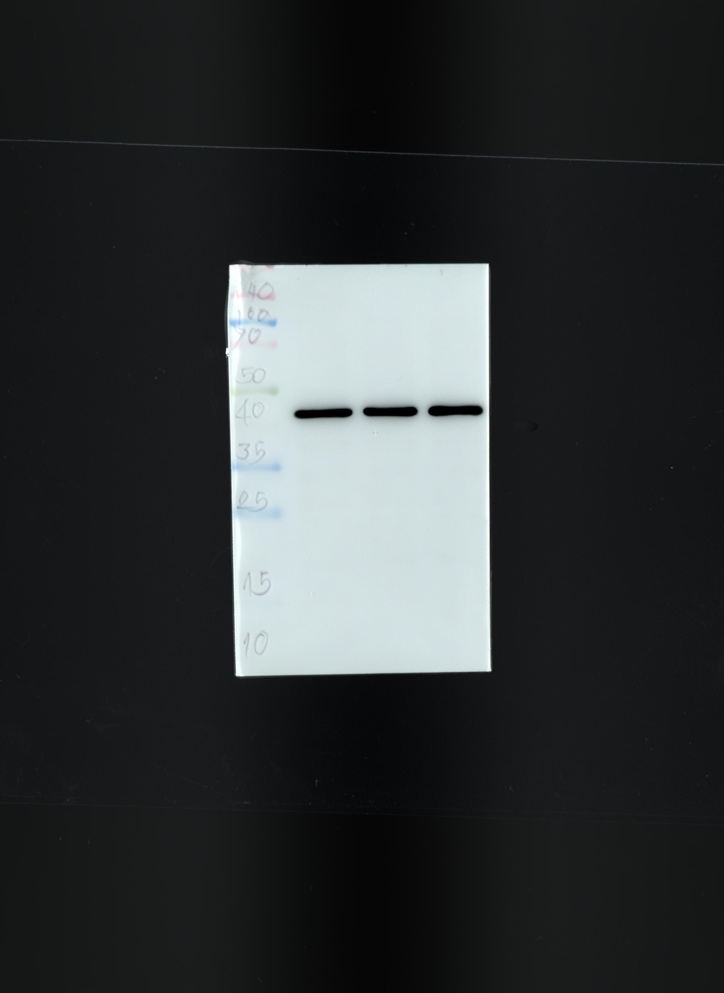 |
| KKU-100 | p53 | 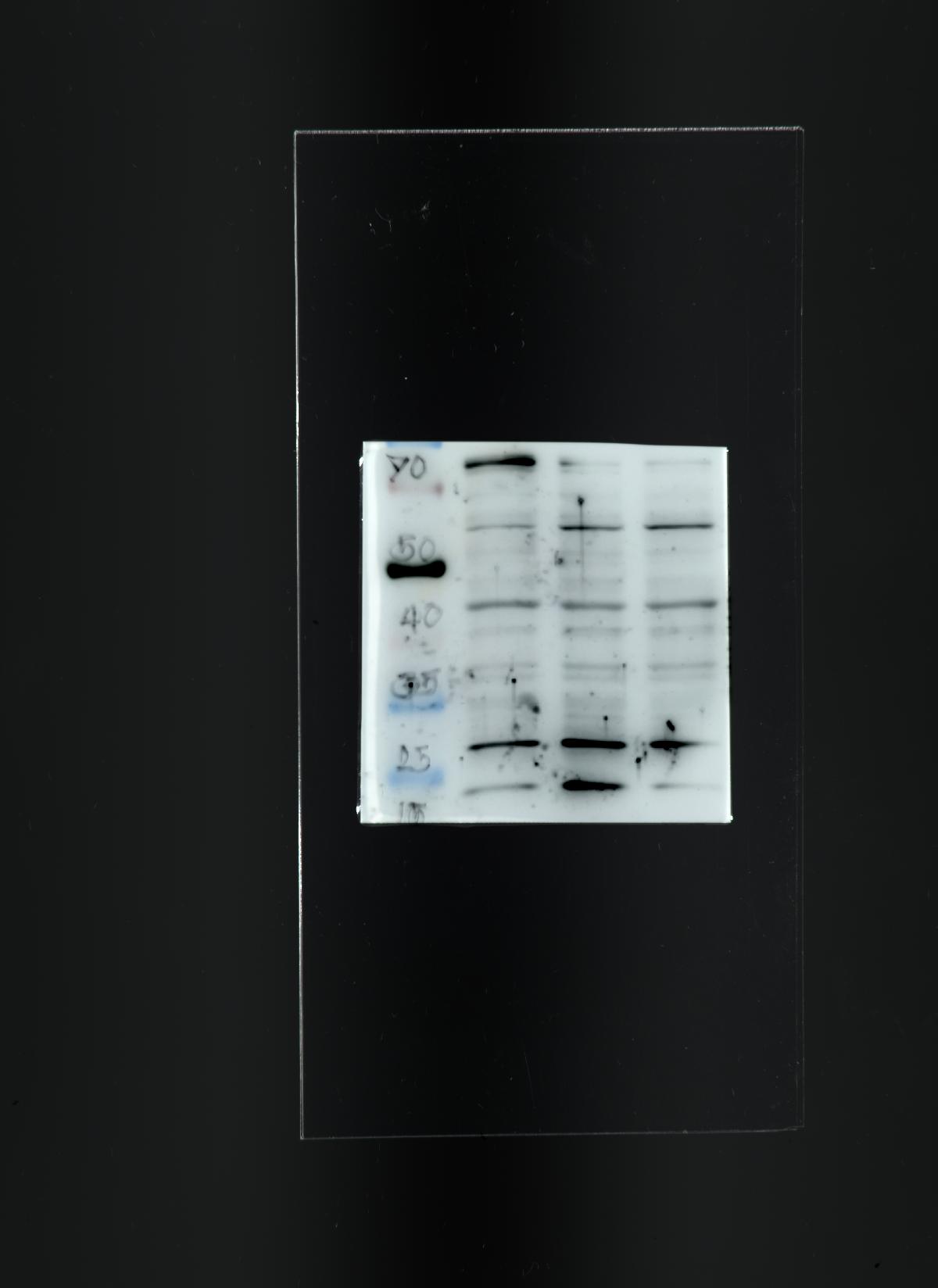 | 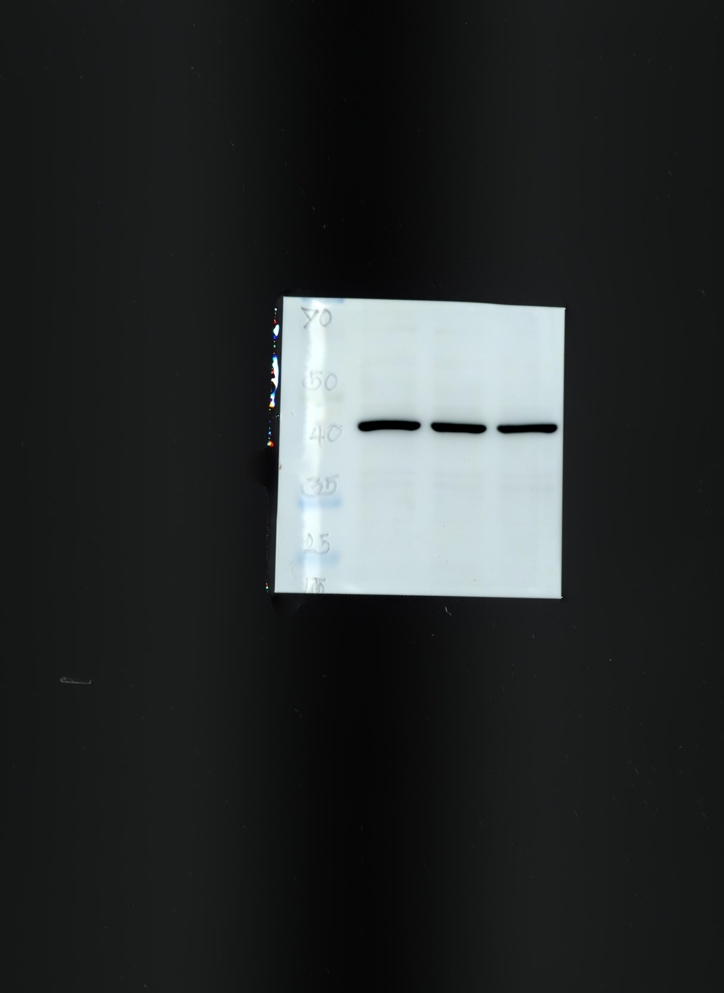 |
| KKU-100 | p21 | 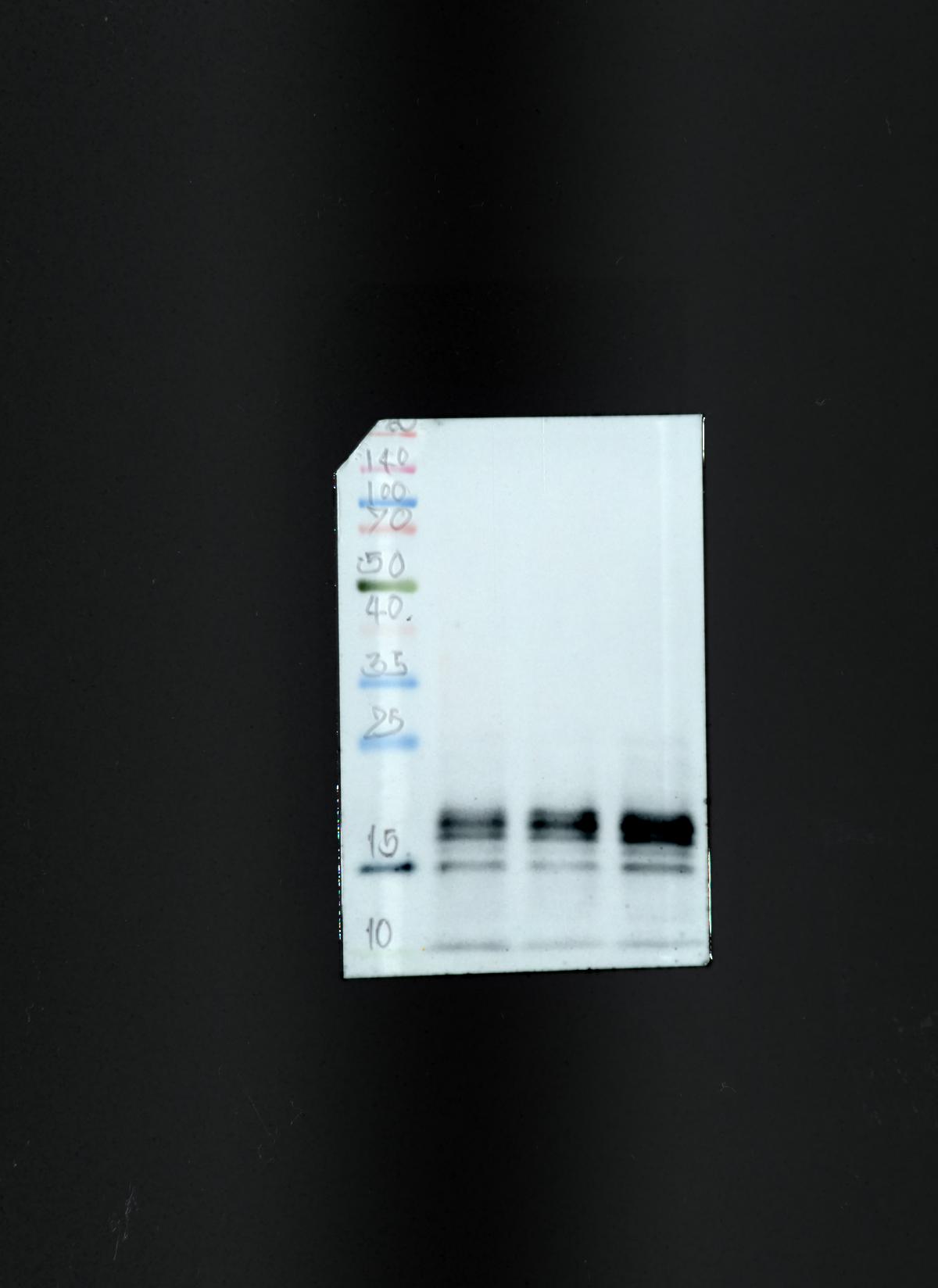 | 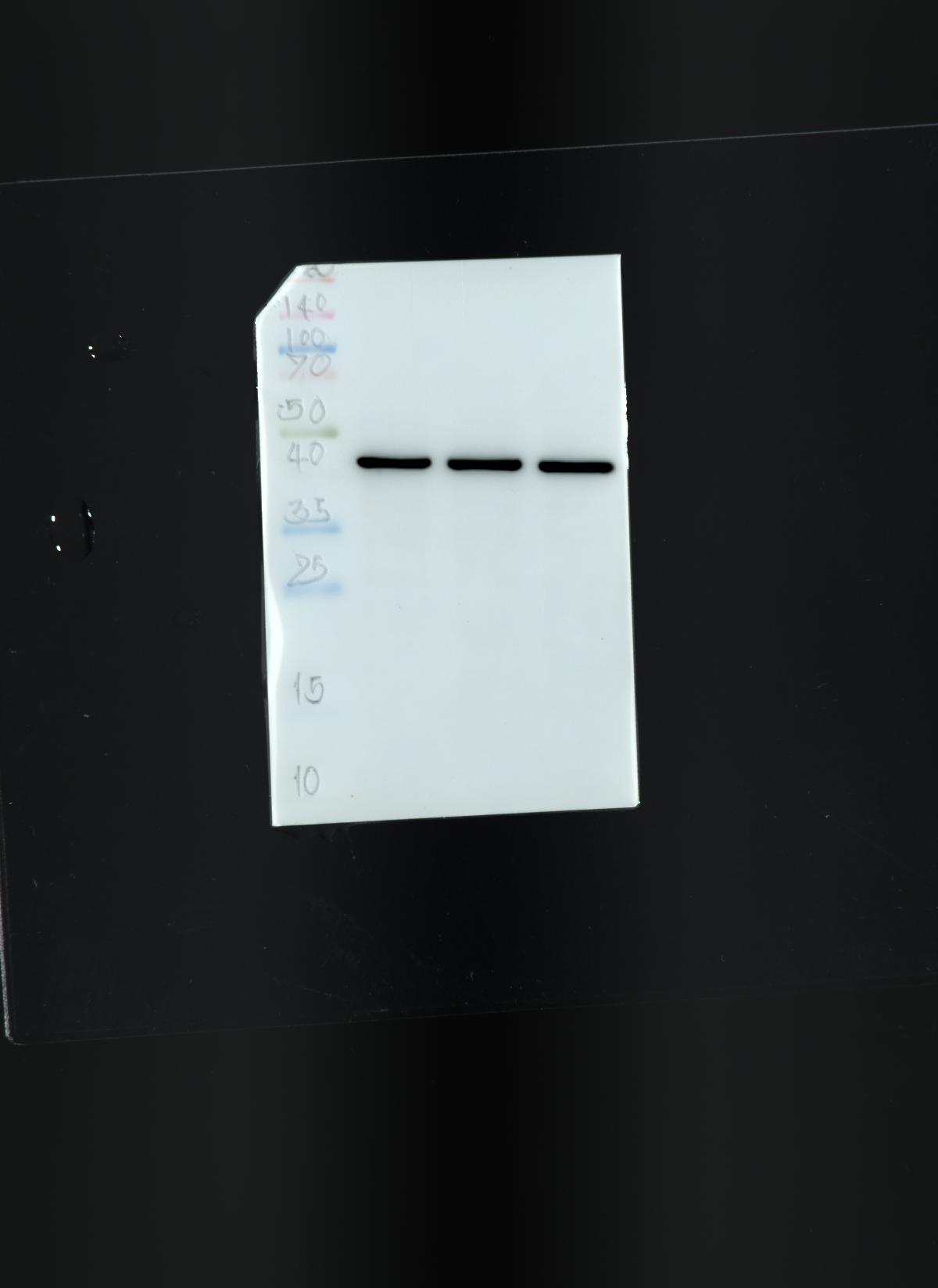 |
